# Restoring Zinc Homeostasis via a Bimetallic Nanozyme to Amplify Ferroptosis and Antitumor Immunity for Prostate Cancer Treatment

**DOI:** 10.34133/bmr.0378

**Published:** 2026-06-26

**Authors:** Jinzhuo Ning, Kun Jiang, Haoyong Li, Fan Cheng

**Affiliations:** Department of Urology, Renmin Hospital of Wuhan University, Wuhan430060, Hubei Province, P.R. China.

## Abstract

Prostate cancer therapy is hindered by treatment resistance and an immunosuppressive microenvironment. To address this, we developed an innovative bimetallic nanozyme, Fe/Zn-CNZ@LOx@PEG, that integrates catalytic activity with metabolic and immune modulation. This nanoplatform executes a novel “metabolic-immuno” co-regulation strategy. Its Fe–Zn catalytic sites drive a lactate-fueled cascade reaction within the acidic tumor milieu, generating a burst of cytotoxic hydroxyl radicals. Simultaneously, released zinc ions rectify tumor zinc deficiency, disrupting cellular metabolism and promoting ferroptosis—an iron-dependent cell death—through glutathione depletion and lipid peroxidation. This ferroptotic cell death, in turn, acts as a potent trigger for immunogenic cell death (ICD), stimulating dendritic cell maturation and cytotoxic T cell infiltration to reverse immunosuppression. Consequently, Fe/Zn-CNZ@LOx@PEG demonstrates potent tumor suppression and effectively enhances the efficacy of anti-PD-1 therapy in a prostate cancer mouse model. This work presents a catalytic nanoreactor that co-regulates metabolism and immunity, offering a robust and synergistic strategy for the ferroptosis-immunotherapy of advanced cancers.

## Introduction

Prostate cancer (PCa), the second most prevalent malignancy in men worldwide, poses persistent clinical challenges due to its high incidence and mortality rates [1,2]. A key factor underlying its aggressiveness and treatment failure is a profoundly immunosuppressive tumor microenvironment (TME), which facilitates immune evasion [3]. While immune checkpoint inhibitors (ICIs) have revolutionized oncology, their efficacy in PCa remains limited, characterized by low response rates and prevalent resistance [4,5]. This underscores the urgent need for novel strategies that can remodel the immunosuppressive TME and synergize with immunotherapy.

Inducing immunogenic cell death (ICD), particularly through pathways like ferroptosis, represents a promising approach to convert “cold” tumors into “hot” ones and potentiate ICI efficacy [6–8]. However, their clinical application faces core challenges: First, the overall response rate (ORR) in most solid tumors remains modest (15% to 25%), with widespread primary or acquired resistance and a lack of reliable predictive biomarkers [9,10]. Second, immune-related adverse events (irAEs) occur in 20% to 32% of patients, affecting multiple organ systems and posing significant safety risks [11]. These limitations underscore the bottlenecks of monotherapies and drive exploration of multimodal combination strategies. Current research prioritizes investigating synergistic interactions between ICIs and emerging modalities like metabolic modulation and ferroptosis induction, representing a pivotal avenue for enhancing antitumor immunity. This trend reflects deepening mechanistic insights into immunotherapy and signals potential paradigm shifts in cancer treatment.

Ferroptosis, an iron-dependent, regulated cell death pathway triggered by aberrant accumulation of lipid peroxides (LPOs), has attracted significant attention for its unique metabolic regulation and synergistic potential with ICIs [12,13]. Studies indicate that enhancing ferroptosis susceptibility can reverse ICI resistance [12,14]. Nevertheless, tumor cells employ adaptive resistance mechanisms—such as GPX4 overexpression, FSP1-CoQ_10_ pathway activation, lipid metabolic reprogramming, and iron homeostasis regulation—to evade ferroptosis [15,16]. This evidence suggests that sensitizing tumors to ferroptosis by targeting key nodes (e.g., GPX4, FSP1, and iron metabolism pathways) is essential for establishing novel immune-metabolic therapeutic paradigms. This research direction expands the biological significance of ferroptosis while providing a new conceptual framework for cancer immunotherapy.

Notably, zinc metabolism emerges as a critical and exploitable nexus linking PCa pathobiology, ferroptosis susceptibility, and immune modulation. Unlike normal prostate epithelia that maintain high zinc levels, malignant cells exhibit a characteristic zinc-depleted state, which is intrinsically linked to metabolic reprogramming and contributes to an immunosuppressive milieu [17]. Zinc homeostasis regulation disrupts the tricarboxylic acid (TCA) cycle by inhibiting mitochondrial aconitase and suppresses SLC7A11 expression via the p53 pathway, synergistically depleting glutathione (GSH) and promoting ferroptosis [18–21]. This discovered “metabolism–ferroptosis axis” provides a novel theoretical basis for PCa treatment and underscores zinc’s critical role in tumor metabolic regulation.

In nanomedicine, functional nanozymes are highly promising due to their ability to mimic natural enzyme activity, superior stability, and low cost. Inspired by metalloenzyme structures, single-atom nanozymes constructed via metal-ligand self-assembly show significant therapeutic potential [22]. Significantly, bimetallic catalysts (e.g., Fe–Zn dual-metal active sites) leverage synergistic effects between adjacent metal atoms to achieve enhanced catalytic performance, making them ideal candidates for high-efficiency anticancer nanozymes [23,24]. This design strategy overcomes the performance limitations of single-metal nanozymes and offers novel multi-target intervention opportunities for cancer therapy.

**Fig. 1.**
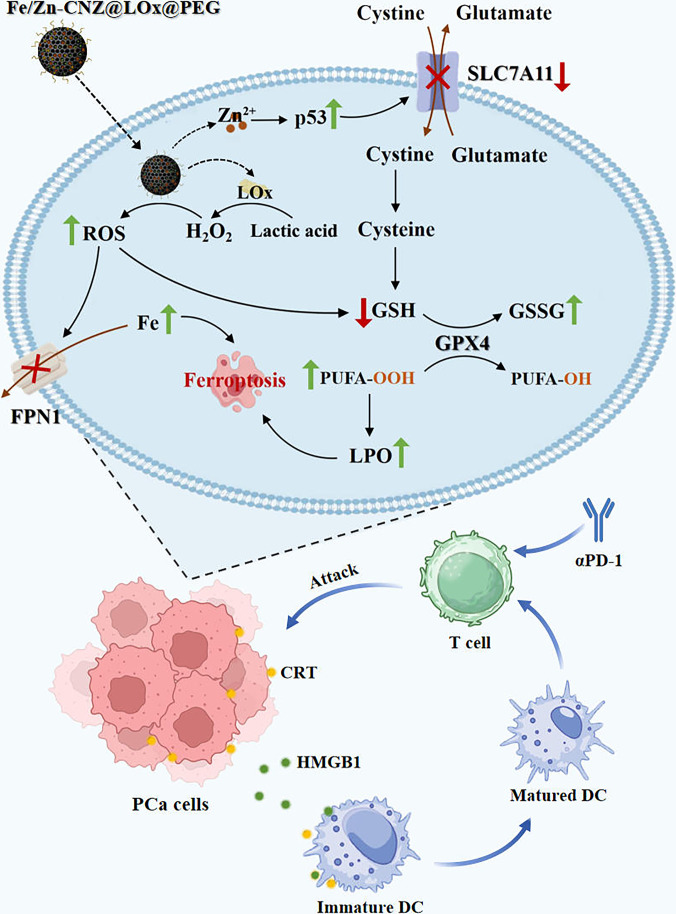
Schematic illustration of the mechanism of Fe/Zn-CNZ@LOx@PEG-mediated ferroptosis activation for evoking systemic tumor immunity in PCa.

In this study, we innovatively developed a type of iron–zinc dual-metal carbon-based nanozyme (CNZ): Fe/Zn-CNZ@LOx@PEG. This system features highly dispersed bimetallic catalytic sites formed through Fe–Zn interfacial interactions. Specifically designed to target the TME, the nanocomposite employs lactate oxidase (LOx) to catalyze the oxidation of intracellular lactate into a substantial amount of H_2_O_2_ [25]. Subsequently, the iron species facilitate the conversion of H_2_O_2_ into highly cytotoxic hydroxyl radicals (·OH) and superoxide anions (·O_2_^−^) while simultaneously depleting GSH to initiate ferroptosis. Concurrently, zinc ions released by the nanoplatform reverse the zinc-deficient state in PCa cells through multi-dimensional mechanisms: (a) inhibiting mitochondrial aconitase to block the TCA cycle and citrate oxidation; (b) activating the p53 pathway to suppress SLC7A11 expression and cooperatively deplete GSH; and (c) reprogramming lipid metabolism to enhance ferroptosis susceptibility. Ferroptosis-induced tumor cell lysis releases tumor-associated antigens (TAAs) and damage-associated molecular patterns (DAMPs), activating dendritic cells (DCs) for antigen presentation and recruiting T cell infiltration (Fig. 1). This process reshapes the immunosuppressive TME via a dual “ablation-immunoactivation” mechanism. Our strategy achieves synergistic metabolic reprogramming and ferroptosis induction, offering a targeted and safe innovative solution for PCa immunotherapy.

The core innovation lies in the spatiotemporal integration of zinc homeostasis restoration, bimetallic nanocatalysis, and ICD, simultaneously overcoming 3 major therapeutic gaps: the singularity of molecular targeting, the low response rates of immunotherapy, and the biological inertia of traditional nanocarriers. This intelligently responsive nanoplatform not only provides a breakthrough strategy for overcoming therapy resistance in advanced PCa but also establishes a novel “metabolic-immuno co-regulation” paradigm.

## Materials and Methods

### Chemicals and reagents

The following chemicals and reagents were used: benzene-1,3,5-tricarboxylic acid (H_3_BTC, 98%), ferric chloride hexahydrate (FeCl_3_·6H_2_O, 98%), iron powder (reduced), N,N-dimethylformamide (DMF, ≥99.5%), hydrofluoric acid (≥40%), nitric acid (60% to 65%), zinc nitrate trihydrate [Zn(NO_3_)_2_·3H_2_O], and deionized (DI) water (all from Sinopharm Chemical Reagent Co. Ltd.); 2ʹ,7ʹ-dichlorodihydrofluorescein diacetate (DCFH-DA) probe kit, poly(ethylene glycol) (PEG; molecular weight ~5,000), Citrate Assay Kit, ATP Assay Kit, Calcein-AM/PI Cell Viability/Cytotoxicity Assay Kit, and JC-1 Mitochondrial Membrane Potential Assay Kit (all from Beyotime Biotechnology); Aconitase Activity Assay Kit (Solarbio Science & Technology Co. Ltd.); LOx (Aladdin Scientific); monoclonal antibodies against GPX4, FPN1 (ferroportin 1), p53, SLC7A11, and glyceraldehyde-3-phosphate dehydrogenase (GAPDH) (all from Wuhan Proteintech Group); CD3, CD8, CD11b, CD80, CD86, and Gr-1 flow cytometry antibodies (Wuhan Proteintech Group); tumor necrosis factor-α (TNF-α), interferon-γ (IFN-γ), interleukin-1β (IL-1β), and IL-10 enzyme-linked immunosorbent assay (ELISA) kits (Beyotime Biotechnology).

### Material synthesis

#### Synthesis of MIL-100(Fe)

Reduced iron powder (0.56 g), H_3_BTC (1.4 g), 40% hydrofluoric acid (500 μl), and 65% nitric acid (475 μl) were added to 50 ml of DI water. The mixture was ultrasonicated for 1 h, then transferred to a Teflon-lined autoclave, and reacted hydrothermally at 150 °C for 12 h. After cooling, the MIL-100(Fe) product was isolated by centrifugation, washed in DI water at 80 °C for 3 h, washed in ethanol at 60 °C for 3 h, and dried overnight at 80 °C. Finally, the sample was dried under vacuum at 150 °C for 12 h to obtain an orange powder.

#### Preparation of iron-based hierarchical porous carbon

MIL-100(Fe) was placed in a tube furnace and carbonized under nitrogen (N_2_) atmosphere with a heating ramp of 2 °C/min to 600 °C, then held at 600 °C for 8 h. After cooling to room temperature under N_2_, the sample was passivated with a N_2_/O_2_ mixture (98%/2%, v/v) for 4 h, yielding PC-Fe (iron-based hierarchical porous carbon). A potassium-promoted catalyst was prepared subsequently via impregnation on PC-Fe. Inductively coupled plasma optical emission spectrometry (ICP-OES) analysis confirmed an Fe content of 15.16 wt %.

#### Preparation of Fe/Zn-CNZ, Fe/Zn-CNZ@PEG, and Fe/Zn-CNZ@LOx@PEG

Fe/Zn-CNZ: An aqueous solution of Zn(NO_3_)_2_·3H_2_O (2 ml, 1 wt %) was added dropwise to PC-Fe (2 g) and allowed to fully impregnate. The sample was dried overnight and then thermally treated at 80 °C under N_2_ flow for 4 h.

Fe/Zn-CNZ@PEG: Fe/Zn-CNZ (10 mg) and PEG (10 mg) were dispersed in DI water (5 ml) and stirred at 37 °C for 24 h. The product was collected by centrifugation (10,000 rpm, 10 min), washed 3 times with DI water, and dried overnight under vacuum.

Fe/Zn-CNZ@LOx@PEG: Fe/Zn-CNZ (10 mg) was dispersed in 1.5 ml of an aqueous solution containing LOx at a concentration of 2 mg/ml and incubated under continuous stirring at 4 °C for 12 h. The resulting product, Fe/Zn-CNZ@LOx, was collected by centrifugation. Subsequently, Fe/Zn-CNZ@LOx (10 mg) was mixed with polyethylene glycol (PEG; molecular weight 4,000 Da; 10 mg) in 5 ml of DI water and stirred at 37 °C for 24 h. The final product, Fe/Zn-CNZ@LOx@PEG, was obtained after repeated washing steps.

### Material characterization

Powder x-ray diffraction (PXRD) patterns were collected on a Bruker D8 Advance diffractometer (Cu Kα radiation, 40 kV, 40 mA) over a 2θ range of 20° to 90° with a step size of 0.01° and a scan rate of 2°/min. Chemical composition was analyzed using ICP-OES (Agilent 700), x-ray fluorescence spectroscopy (XRF; Shimadzu EDX-LE), and scanning electron microscopy (SEM; Hitachi SU-8010). High-resolution transmission electron microscopy (HR-TEM) and elemental mapping were performed on a Titan Themis-Z microscope (Thermo Fisher Scientific). Nitrogen physisorption was measured at −196 °C using a Micromeritics ASAP 2460 analyzer. Specific surface area was calculated using the Brunauer–Emmett–Teller (BET) method, total pore volume was measured at *P*/*P*_0_ = 0.99, and micropore characteristics were determined via the *t*-plot method. X-ray photoelectron spectroscopy (XPS) was performed on a Thermo Scientific ESCALAB 250 XI spectrometer using Al Kα radiation (1,486.6 eV, 150 W); binding energies were referenced to the C1s peak at 284.8 eV. Fourier transform infrared (FT-IR) spectra were collected on a Thermo Scientific Nicolet iS10 spectrometer from 400 to 4,000 cm^−1^ using KBr pellets.

### Zn^2+^ release measurement

Fe/Zn-CNZ@LOx@PEG was incubated in phosphate-buffered saline (PBS; pH 7.4) under stirring at 37 °C. Aliquots were collected at predetermined time points and centrifuged (12,000 rpm, 10 min), and the supernatants were analyzed for released Zn^2+^ concentration using inductively coupled plasma mass spectrometry (ICP-MS) and high-performance liquid chromatography (HPLC).

### H_2_O_2_ and hydroxyl radical (OH) detection

The capacity of various nanoparticles to generate H_2_O_2_ was evaluated using a hydrogen peroxide quantification kit in PBS solutions at different pH levels.

Peroxidase (POD)-like activity was assessed via 3,3′,5,5′-tetramethylbenzidine (TMB) colorimetric assay. The reaction mixture in PBS (pH 5.5) contained H_2_O_2_ (10 mM final concentration) and TMB (0.1 mg/ml). The reaction was initiated by adding PC-Fe@PEG, Fe/Zn-CNZ@PEG, or Fe/Zn-CNZ@LOx@PEG. The absorbance change at 652 nm was monitored over time using an ultraviolet–visible (UV–Vis) spectrophotometer (Thermo Scientific Evolution 220).

### MyC-CaP cell culture and viability assay

Mouse-derived prostate adenocarcinoma MyC-CaP cells were cultured in RPMI 1640 medium supplemented with 10% fetal bovine serum (FBS), 100 U/ml penicillin, and 0.1 mg/ml streptomycin at 37 °C under 5% CO_2_. After seeding onto plates and overnight incubation, cells were treated with PC-Fe@PEG, Fe/Zn-CNZ@PEG, or Fe/Zn-CNZ@LOx@PEG. Cell viability was assessed using a Cell Counting Kit-8 (CCK-8) kit (absorbance measured at 450 nm) and a Calcein-AM/PI Live/Dead Cell Staining Kit observed by confocal microscopy (Zeiss LSM 800).

### C4-2B cells and BPH1 cell culture

The human PCa cell line C4-2B and the benign prostatic hyperplasia epithelial cell line BPH1 were cultured in Dulbecco’s modified Eagle’s medium (DMEM) supplemented with 10% FBS, 100 U/ml penicillin, and 0.1 mg/ml streptomycin, and maintained at 37 °C in a humidified atmosphere containing 5% CO_2_. Following digestion, intracellular Zn^2+^ concentration was analyzed by ICP-MS in combination with HPLC.

### Transwell cell migration assay

Transwell inserts (8-μm pore, Corning) were placed in 24-well plates. The lower chamber was filled with complete medium containing chemoattractant (e.g., 10% FBS in RPMI). A suspension of MyC-CaP cells was added to the upper chamber. Plates were incubated for 24 h at 37 °C under 5% CO_2_. Medium and nonmigrated cells in the upper chamber were gently removed using a cotton swab and PBS washes. Migrated cells adhering to the lower membrane surface were fixed with 4% paraformaldehyde (PFA) for 15 min and stained with 1% crystal violet solution for 20 min. Membranes were excised and mounted on slides, and migrated cells were imaged and counted using an optical microscope (Zeiss Axio Observer).

### Intracellular zinc content measurement

MyC-CaP cells (1 × 10^5^ cells per well) were seeded in 6-well plates for 24 h. Cells were then treated with different concentrations of nanoparticles (dispersed in PBS) for 6 h. After incubation, cells were washed twice with PBS and intracellular zinc was detected via flow cytometry (BD LSRFortessa X-20) using a zinc-specific fluorophore (as per kit protocol). For quantitative Zn^2+^ determination, treated cells were harvested and lysed with aqua regia (3:1 HCl:HNO_3_), and Zn content was measured via ICP-MS.

### GSH depletion assay

In vitro: PBS solutions containing PC-Fe@PEG, Fe/Zn-CNZ@PEG, or Fe/Zn-CNZ@LOx@PEG were mixed with GSH solution (1 mM) and incubated at 37 °C for 1 h. The mixture was centrifuged (10,000 rpm, 10 min). Residual GSH in the supernatant (0.5 ml) was reacted with 5,5′-dithiobis(2-nitrobenzoic acid) (DTNB; Ellman’s reagent) solution under dark incubation (e.g., 10 to 15 min). The absorbance change at 412 nm was measured to calculate the concentration of remaining GSH, indicating depletion capacity.

Intracellular: MyC-CaP cells were co-incubated with nanoparticles for 24 h. Cells were harvested, sonicated (300 W, 30 min), and centrifuged. The supernatant was analyzed for intracellular GSH concentration using the GSH assay kit according to the manufacturer’s protocol.

### Intracellular reactive oxygen species detection

Intracellular reactive oxygen species (ROS) levels were detected using the DCFH-DA fluorescent probe. MyC-CaP cells (5 × 10^4^ cells per well) were seeded in 6-well plates and treated with nanoparticles for 24 h. Cells were then stained with 50 μM DCFH-DA working solution for 30 min at 37 °C in the dark. After PBS washes, fluorescence intensity (excitation: 488 nm, emission: 525 nm) was quantified via flow cytometry (BD LSRFortessa X-20).

### Mitochondrial membrane potential (ΔΨm) measurement

MyC-CaP cells in 6-well plates were treated with nanoparticles for 24 h. After removing the medium, cells were stained with JC-1 working solution (prepared as per kit instructions) and incubated at 37 °C in the dark for 20 min. Cells were washed with PBS, and the shift in red (aggregates, ~590-nm emission) to green (monomers, ~530-nm emission) fluorescence ratio, indicative of mitochondrial membrane potential (MMP) depolarization, was observed immediately using a fluorescence microscope (Zeiss Axio Imager) or quantified using a microplate reader.

### Citrate, aconitase activity, and ATP detection

MyC-CaP cells treated with nanoparticles for 24 h were processed. Intracellular citrate concentration and aconitase activity were measured using specific assay kits according to the manufacturers’ protocols (Beyotime Citrate Assay Kit, Solarbio Aconitase Activity Kit). Adenosine triphosphate (ATP) levels were quantified using the ATP assay kit (Beyotime, based on a luciferin–luciferase reaction). Absorbance/chemiluminescence was recorded using a microplate reader.

### ICD marker detection

MyC-CaP cells treated with nanoparticles for 24 h were fixed. Surface exposure of calreticulin (CRT) and extracellular release of high-mobility group box 1 (HMGB1) protein were assessed by immunofluorescence staining using specific primary antibodies (anti-CRT, anti-HMGB1) followed by fluorescently labeled secondary antibodies. Staining patterns were visualized and imaged using a fluorescence microscope or confocal laser scanning microscope (Zeiss LSM 800).

### Maturation of DCs induced by ICD

MyC-CaP cells pretreated with nanoparticles (to induce ICD) were cocultured with immature bone marrow-derived dendritic cells (BMDCs) at a defined ratio (e.g., 5:1) for 48 h. BMDCs were collected, stained with fluorescence-conjugated antibodies against CD11c, CD80, and CD86, and analyzed by flow cytometry (BD LSRFortessa X-20). Up-regulation of costimulatory molecules CD80 and CD86 on CD11c^+^ cells indicated BMDC maturation capacity triggered by ICD.

### Western blot analysis

Cells were lysed in radioimmunoprecipitation assay (RIPA) buffer containing protease inhibitors. Protein concentration was determined using a bicinchoninic acid (BCA) assay. Equal amounts of protein were separated by sodium dodecyl sulfate–polyacrylamide gel electrophoresis (SDS-PAGE) and transferred onto polyvinylidene difluoride (PVDF) membranes. Membranes were blocked with 5% nonfat milk or bovine serum albumin (BSA) in tris-buffered saline with Tween 20 (TBST) and then probed overnight at 4 °C with specific primary antibodies (anti-GPX4, anti-FPN1, anti-p53, anti-SLC7A11, anti-GAPDH, etc.). After washing, membranes were incubated with appropriate horseradish peroxidase (HRP)-conjugated secondary antibodies. Protein bands were visualized using enhanced chemiluminescence (ECL) substrate, and band intensities were quantified using ImageJ software (National Institutes of Health).

### Subcutaneous tumor model

All animal procedures were approved by the Institutional Animal Care and Use Committee (IACUC) of Renmin Hospital of Wuhan University (approval no. WDRM20230505A) and complied with Chinese national regulations. Subcutaneous MyC-CaP tumors were established in the dorsal flank of male C57BL/6 mice (6 to 8 weeks old) by injecting 100 μl of cell suspension (5 × 10^6^ cells/ml). When tumor volumes reached approximately 50 to 100 mm^3^, mice were randomly assigned to 4 groups (*n* = 5 per group):

1. Control: Intravenous injection of sterile PBS (100 μl).
2. αPD-1 monotherapy: Intraperitoneal injection of anti-PD-1 antibody (αPD-1, clone RMP1-14, BioXCell, 0.8 mg/kg) every 3 d, for a total of 3 doses.
3. Fe/Zn-CNZ@LOx@PEG monotherapy: Intravenous injection of Fe/Zn-CNZ@LOx@PEG (1.5 mg Fe/kg dispersed in PBS, 100 μl) every 3 d, for a total of 3 doses.
4. Combination therapy (Fe/Zn-CNZ@LOx@PEG + αPD-1): Intravenous injection of Fe/Zn-CNZ@LOx@PEG (1.5 mg Fe/kg) plus intraperitoneal injection of αPD-1 (0.8 mg/kg) every 3 d, for a total of 3 doses. Tumor volume [*V* = (Length × Width^2^)/2] was measured every 3 d using digital calipers.

### Bilateral tumor model

MyC-CaP cells (5 × 10^6^ cells/ml, 100 μl) were injected subcutaneously into the left dorsal flank of C57BL/6 mice to establish the primary tumor. Two days later, an identical injection was performed in the right dorsal flank to establish the distant tumor. When primary tumors reached ~50 to 100 mm^3^, mice were randomized into 4 groups (*n* = 5 per group):

1. Control: Untreated.
2. αPD-1 monotherapy: Intraperitoneal injection of αPD-1 (0.8 mg/kg) every 3 d, 3 doses total.
3. Fe/Zn-CNZ@LOx@PEG monotherapy: Intratumoral injection of Fe/Zn-CNZ@LOx@PEG (0.3 mg/ml, 50 μl) into the primary tumor every 3 d, 3 doses total.
4. Combination therapy (Fe/Zn-CNZ@LOx@PEG + αPD-1): Intratumoral injection of Fe/Zn-CNZ@LOx@PEG (0.3 mg/ml, 50 μl) into the primary tumor plus intraperitoneal injection of αPD-1 (0.8 mg/kg) every 3 d, 3 doses total. Volumes of both primary and distant tumors were measured every 3 d [*V* = (Length × Width^2^)/2].

### In vivo immune response analysis

Tumor-bearing mice were euthanized at defined endpoints. Draining lymph nodes (LNs) and spleens were harvested. Single-cell suspensions were prepared:

DCs: Tumor-draining LNs were processed. CD11c^+^ cells were analyzed for expression of maturation markers (CD80^+^, CD86^+^) via flow cytometry.

CD8^+^ T cells: Splenocytes were stained with anti-CD3 and anti-CD8. CD8^+^ T cell population frequency and activation were assessed by flow cytometry.

Tumor-infiltrating lymphocytes (TILs): Excised tumors were dissociated into single-cell suspensions. TILs, including CD45^+^, CD3^+^CD4^+^, and CD3^+^CD8^+^, and myeloid-derived suppressor cells (MDSCs; CD11b^+^Gr-1^+^) were quantified by flow cytometry.

Cytokines: Serum or tumor homogenates were analyzed for cytokine levels (TNF-α, IFN-γ, IL-1β, IL-10) using ELISA kits.

### Systemic toxicity evaluation

Mice received an intravenous injection of Fe/Zn-CNZ@LOx@PEG (dose equivalent to therapeutic dose). One week post-injection, blood samples were collected via retro-orbital bleeding or cardiac puncture:

Liver/kidney function: Serum levels of aspartate aminotransferase (AST), alanine aminotransferase (ALT), blood urea nitrogen (BUN), and creatinine (Cr) were measured using standard clinical analyzers.

Hematology: Complete blood count (CBC), including white blood cells (WBCs), red blood cells (RBCs), hemoglobin (HGB), and platelets (PLTs), was performed. Mice were then euthanized. Major organs (heart, liver, spleen, lungs, kidneys) were harvested, fixed in 4% PFA, embedded in paraffin, sectioned (5 μm), and stained with hematoxylin and eosin (H&E). Histopathological analysis was conducted by a blinded pathologist using light microscopy (Zeiss Axio Imager).

### Statistical analysis

All data are presented as mean ± standard deviation (SD) from at least 3 independent experiments. Statistical analysis was performed using GraphPad Prism software (version 8.0) and SPSS (IBM version 21.0). Differences between 2 groups were analyzed using a 2-tailed unpaired Student’s *t* test. Comparisons among multiple groups were performed using one-way analysis of variance (ANOVA) followed by post hoc tests (e.g., Tukey’s or Bonferroni’s test). A *P* value of <0.05 was considered statistically significant, and a *P* value of <0.01 was considered highly statistically significant.

## Results and Discussion

### Synthesis and characterization of Fe/Zn-CNZ@PEG

**Fig. 2.**
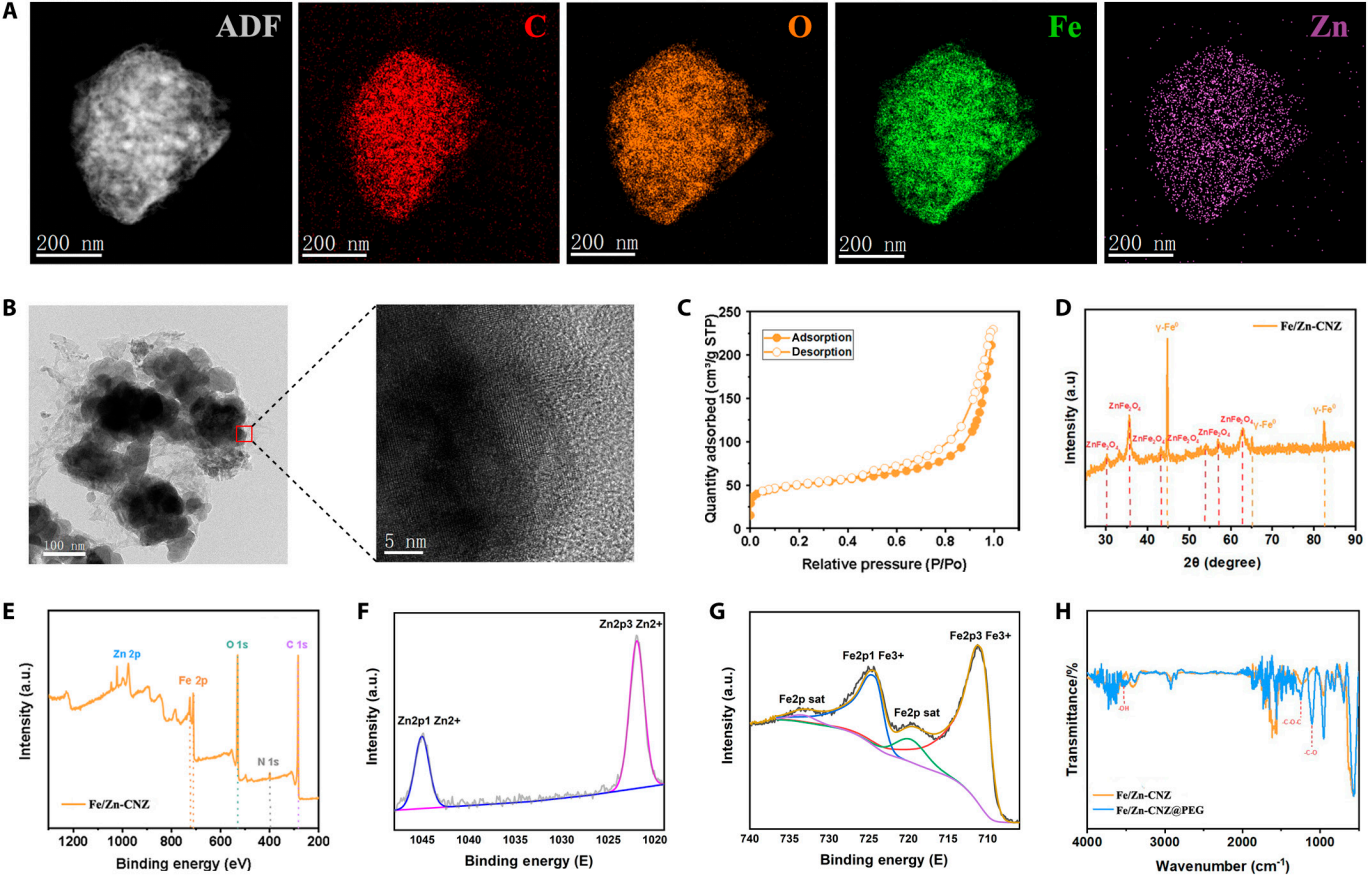
Materials characterization for nanoparticles. (A) Element mappings and (B) HR-TEM images of Fe/Zn-CNZ. (C) N_2_ physisorption analysis of Fe/Zn-CNZ. (D) XRD patterns of Fe/Zn-CNZ. (E) XPS spectrums of Fe/Zn-CNZ. High-resolution XPS spectra of the (F) Zn 2p peaks and (G) Fe 2p peaks. (H) FT-IR spectrums of Fe/Zn-CNZ and Fe/Zn-CNZ@PEG.

The Fe/Zn-CNZ@PEG composite was successfully synthesized through pyrolysis of the MIL-100(Fe) precursor, followed by zinc introduction via an impregnation method and subsequent coating with polyethylene glycol (PEG) to enhance biocompatibility (Fig. 2A and B). This material serves as the functional carrier for the Fe/Zn-CNZ@LOx@PEG composite nanozyme, with its high specific surface area and hierarchically porous structure being critical for catalytic performance.

The energy-dispersive x-ray spectroscopy (EDS) elemental mapping images (Fig. 2A) clearly demonstrate the homogeneous distribution of Fe, Zn, C, and O throughout the material, indicating the successful incorporation of zinc into the carbon–nitrogen framework without significant agglomeration. This confirms the excellent compositional uniformity of the composite. Transmission electron microscopy (TEM) images (Fig. 2B) reveal that Fe/Zn–CNZ exhibits a well-defined flower- or urchin-like morphology, composed of self-assembled primary particles several tens of nanometers in size, coated with an amorphous carbon layer, indicating high structural regularity and good dispersibility. N_2_ adsorption–desorption isotherm analysis (Fig. 2C) shows a typical type IV curve with an H3 hysteresis loop, confirming the hierarchical porous structure of the material, dominated by mesopores. This porous architecture facilitates mass transport and exposes catalytic sites, providing an ideal substrate for subsequent enzyme loading and catalytic reactions. The specific surface area and pore structure parameters further indicate that the introduction of zinc did not significantly compromise the porosity of the carbon framework, suggesting a high degree of dispersion of the metal species. The x-ray diffraction (XRD) pattern (Fig. 2D) displays distinct diffraction peaks at 35.5°, 43.2°, and 62.6°, which are assigned to the crystal planes of γ-Fe_2_O_3_ and zinc ferrite (ZnFe_2_O_4_). These results confirm the successful incorporation of zinc and the formation of a stable composite metal oxide phase with iron. The full-scan XPS spectra (Fig. 2E to G) show characteristic photoelectron peaks of Fe 2p, Zn 2p, O 1s, and C 1s, further verifying the presence of these elements and the surface composition, consistent with the EDS findings. After calibration with the C–C/C–H peak at 284.8 eV, the Zn 2p spectrum showed 2 peaks at 1,021.93 and 1,045.03 eV, corresponding to Zn 2p^3/2^ and Zn 2p^1/2^, respectively, indicating that Zn was mainly present as Zn^2+^. Meanwhile, the Fe 2p spectrum displayed 2 peaks at 710.84 and 724.25 eV, assigned to Fe 2p^3/2^ and Fe 2p^1/2^, respectively. Together with the characteristic satellite peak features, these results indicate that Fe was predominantly present in the Fe^3+^ state (Fig. 2F and G).

Furthermore, FT-IR spectroscopy was employed to characterize the PEG modification (Fig. 2H). Compared with the unmodified Fe/Zn–CNZ, the Fe/Zn–CNZ@PEG composite exhibits new absorption bands at around 1,100 and 3,400 cm^−1^, attributed to C–O–C stretching vibrations and –OH groups, respectively. This confirms the successful coating of PEG on the nanoparticle surface, enhancing the hydrophilicity and biocompatibility of the material. In summary, the Fe/Zn–CNZ@PEG composite possesses a high specific surface area, hierarchical porosity, excellent elemental homogeneity, and effective surface modification, providing a solid material foundation for its application as a stable and efficient multifunctional nanozyme in tumor-targeting therapeutic systems.

Dynamic light scattering (DLS) analysis (Fig. S1) revealed that the hydrodynamic diameter (Dh) of Fe/Zn-CNZ@LOx@PEG was approximately 220.19 nm. After treatment with laccase (LOx) and polyethylene glycol (PEG) coating, the Dh of the nanoparticles increased only slightly. Zeta potential measurements (Fig. S2) indicated that the potential of Fe/Zn-CNZ and Fe/Zn-CNZ@LOx@PEG increased from −23.7 mV to 14 mV, suggesting successful loading of PEG and LOx and improved dispersion stability (Fig. S3).

### Catalytic performance and chemodynamic properties

**Fig. 3.**
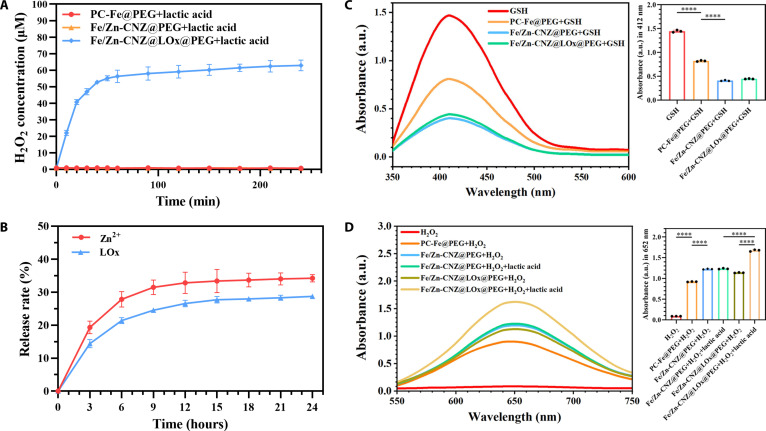
Catalytic performance and chemodynamic properties. (A) The H_2_O_2_ level was catalyzed by different groups (*n* = 3). (B) Zn^2+^ and LOx release curves of Fe/Zn-CNZ@LOx@PEG over time (*n* = 3). (C) UV–Vis–NIR (near-infrared) absorbance of GSH degradation. (D) UV–Vis absorption spectra of the catalyzed oxidation of TMB as catalyzed by different groups. ****P < 0.0001.

Although H_2_O_2_ levels are generally elevated in tumor cells compared to normal cells, the absolute concentration remains insufficient to support efficient catalytic therapy, representing a major limitation for nanozyme-mediated treatment [26]. To address this challenge, we developed a self-supplying H_2_O_2_ cascade system based on lactate oxidation, designated as Fe/Zn-CNZ@LOx@PEG, and evaluated its catalytic performance and antitumor efficacy in comparison with its precursor materials, PC-Fe@PEG and Fe/Zn-CNZ@PEG. As shown in Fig. 3A, Fe/Zn-CNZ@LOx@PEG continuously and efficiently generated H_2_O_2_ in the presence of lactate, with a significantly higher yield than control materials (PC-Fe@PEG and Fe/Zn-CNZ@PEG), demonstrating its superior capability for in situ H_2_O_2_ production and potential to alleviate H_2_O_2_ deficiency in the TME. To simulate the behavior of the nanozyme in tumor tissue, we investigated the release profiles of Zn^2+^ and LOx in PBS at pH 6.5, mimicking the acidic TME. As depicted in Fig. 3B, the cumulative release rates of Zn^2+^ and LOx reached 34.2% and 28.7% within 24 h, respectively, indicating sustained and controllable release behavior beneficial for long-term catalytic reactions and ion-mediated therapeutic effects. We further systematically evaluated the chemodynamic properties of Fe/Zn-CNZ@LOx@PEG. The GSH depletion capability was assessed using the DTNB assay (Fig. 3C). The absorbance at 412 nm decreased most significantly in the Fe/Zn-CNZ@LOx@PEG and Fe/Zn-CNZ@PEG groups compared to PC-Fe@PEG, confirming efficient GSH consumption, which can disrupt the antioxidant defense of tumor cells and enhance oxidative stress. Moreover, the POD-like activity was evaluated via the TMB colorimetric reaction. Incubation of Fe/Zn-CNZ@LOx@PEG (20 μg/ml) with H_2_O_2_, lactate, and TMB at pH 5.5 for 600 s resulted in a pronounced absorption peak at 652 nm, which was significantly stronger than that of the lactate-free control group (Fig. 3D). This indicates that Fe/Zn-CNZ@LOx@PEG efficiently decomposes lactate to generate H_2_O_2_, which is subsequently catalyzed to produce ·OH radicals, thereby oxidizing TMB. Quantitative analysis further confirmed its remarkable POD-like activity. Finally, in an acidic simulated environment, we measured the lactate catalytic performance of Fe/Zn-CNZ@LOx@PEG using the colorimetric method (Fig. S4). The results showed that its Michaelis constant (*K*_m_) was 3.33 ± 0.3 mM, and the maximum reaction rate (*V*_max_) was 0.67 ± 0.05 μM/s. This indicates that its efficiency was good under these conditions. Overall, Fe/Zn-CNZ@LOx@PEG exhibits notable self-supplying H_2_O_2_ capability, controllable release kinetics, efficient GSH depletion, and outstanding POD activity in a lactate-rich environment. These properties collectively contribute to its superior chemodynamic therapeutic performance, highlighting its strong potential for tumor-targeted therapy.

### Fe/Zn-CNZ@LOx@PEG inhibits PCa cell viability and migration

**Fig. 4.**
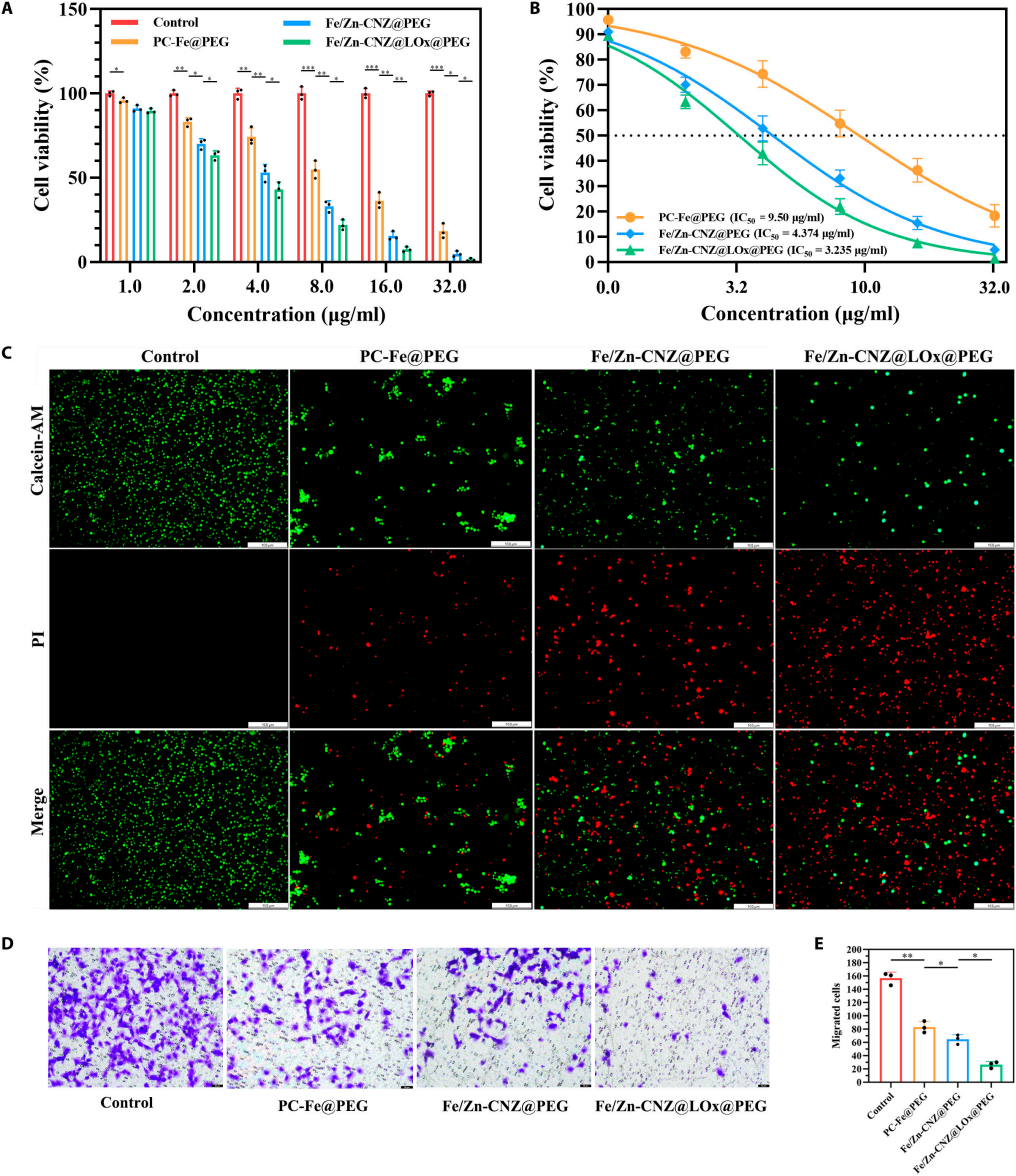
In vitro anti-PCa efficacy of Fe/Zn-CNZ@LOx@PEG. (A) Cell viability of MyC-CaP cells following treatment with escalating concentrations of PC-Fe@PEG, Fe/Zn-CNZ@PEG, or Fe/Zn-CNZ@LOx@PEG (*n* = 3). (B) Calculated IC_5__0_ values for both nanomaterials. (C) Fluorescent microscopy images of MyC-CaP cells costained with Calcein-AM (live cells, green) and propidium iodide (dead cells, red) across treatment groups. Scale bar, 100 μm. (D) Transwell migration assay assessing invasive capacity of MyC-CaP cells under different treatment conditions (*n* = 3). (E) Quantitative analysis of migrated cell counts. *P < 0.05, **P < 0.01, ***P < 0.001.

Fe/Zn-CNZ@LOx@PEG exhibits significant cellular uptake ability toward MyC-CaP cells (Fig. S5) and shows obvious dose-dependent cytotoxicity.. As shown in Fig. 4A and B, the nanomaterial induced near-complete cell death at 32 μg/ml, with a calculated half-maximal inhibitory concentration (IC_50_) of 3.235 μg/ml. Live/Dead staining further validated that at the 3.235 μg/ml dose (preserving 50% cell viability), Fe/Zn-CNZ@LOx@PEG induced substantially higher cell death compared to PC-Fe@PEG and Fe/Zn-CNZ@PEG (Fig. 4C), visualized as extensive red fluorescence labeling of dead cells. In Transwell migration assays, Fe/Zn-CNZ@LOx@PEG also demonstrated superior inhibition of MyC-CaP cell migration relative to PC-Fe@PEG and Fe/Zn-CNZ@PEG (Fig. 4D and E), indicating that zinc incorporation not only enhances cytotoxicity but also effectively suppresses tumor invasive and metastatic potential.

### Fe/Zn-CNZ@LOx@PEG induces oxidative stress in PCa cells

**Fig. 5.**
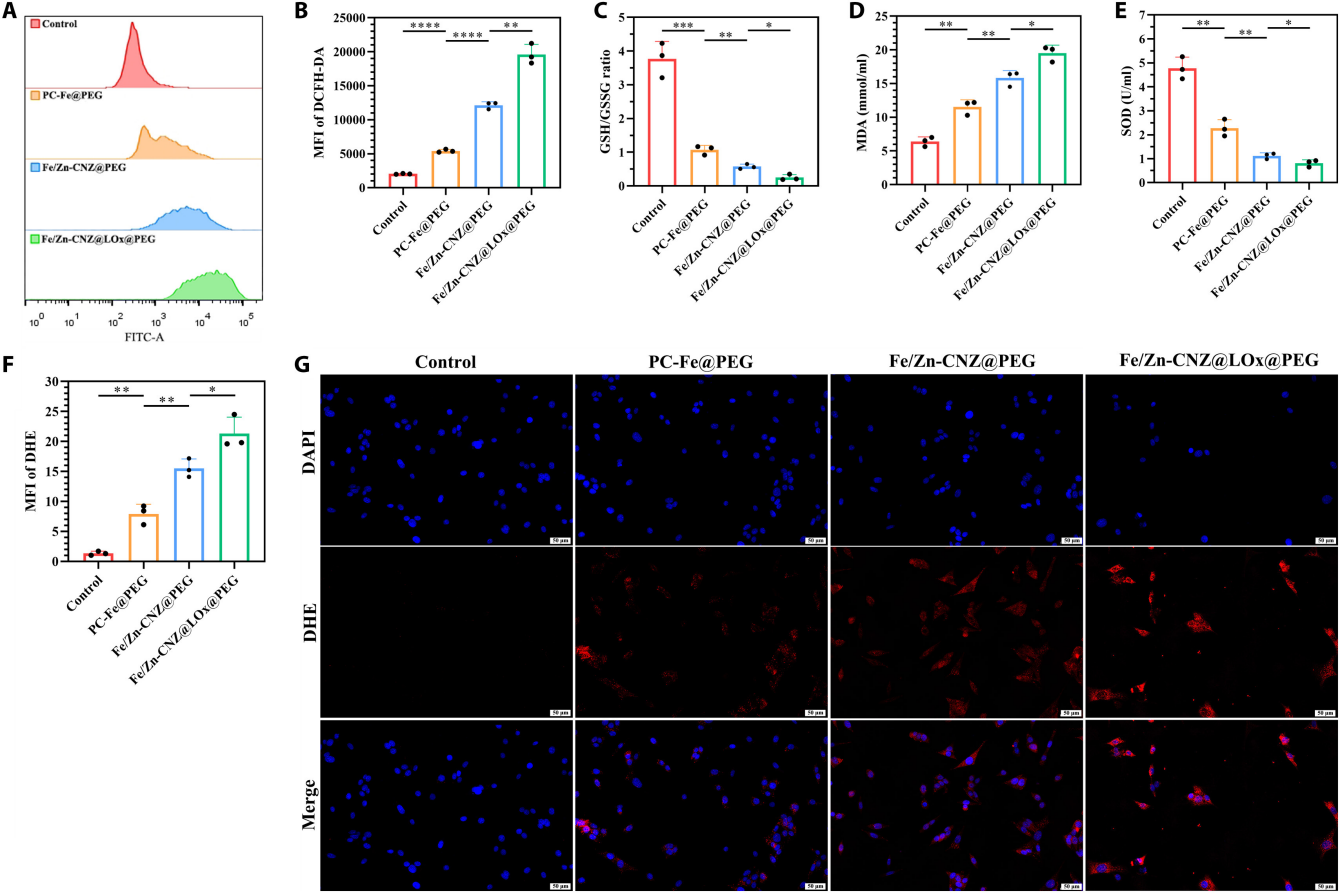
Oxidative stress induction by Fe/Zn-CNZ@LOx@PEG in PCa cells. (A) ROS levels in MyC-CaP cells detected using DCFH-DA fluorescent probe. (B) Quantitative analysis of DCFH-DA fluorescence intensity (*n* = 3). (C) GSH/GSSG ratio measurements across treatment groups (*n* = 3). (D) MDA content as lipid peroxidation marker (*n* = 3). (E) SOD activity in different groups (*n* = 3). (G) Fluorescent imaging of ROS using DHE probe (scale bar, 50 μm) and (F) corresponding quantitative analysis. *P < 0.05, **P < 0.01, ***P < 0.001, ****P < 0.0001.

Leveraging the intrinsic POD-like catalytic activity of Fe/Zn-CNZ@LOx@PEG, we systematically investigated its role in regulating PCa oxidative stress networks. The DCFH-DA probe is used to detect the intracellular ROS content. The data show that Fe/Zn-CNZ@LOx@PEG induced significantly greater intracellular ROS bursts than PC-Fe@PEG and Fe/Zn-CNZ@PEG (Fig. 5A and B). This exponential amplification of ROS stems from the synergistic effect of a triple catalytic pathway: (a) LOx catalyzes the oxidation of intracellular lactate to generate substantial amounts of H_2_O_2_; (b) the Fe^3+^/Fe^2+^ redox cycle converts H_2_O_2_ into hydroxyl radicals (·OH) via the classical Fenton reaction; and (c) consistent with reported synergistic effects in bimetallic systems [24,27], the introduction of Zn^2+^ is proposed to enhance the POD-like activity, potentially by modulating the electronic structure of iron and stabilizing high-valent iron intermediates (e.g., Fe^4+^=O), thereby accelerating ROS generation kinetics. This proposed “zinc-augmented Fenton synergy” is supported by the enhanced catalytic activity of the Fe/Zn-CNZ@PEG system in the TMB oxidation assay (Fig. 3D) compared to the Zn-free control, and aligns with literature on the role of secondary metals in improving Fenton reaction efficiency.

Concomitant antioxidant system collapse further intensified oxidative stress imbalance. DTNB assays revealed rapid depletion of GSH thiol groups (-SH) by Fe/Zn-CNZ@LOx@PEG, leading to exhaustion of this key reducing agent reservoir. Crucially, the reduced/oxidized glutathione (GSH/GSSG) ratio—a core oxidative stress biomarker—showed significant reduction in Fe/Zn-CNZ@LOx@PEG-treated cells (Fig. 5C). This ratio decline directly reflects cellular redox homeostasis collapse, as accumulating GSSG (the dimeric product of oxidized GSH) signals decompensation of antioxidant defenses.

The terminal effects of oxidative stress manifested as irreversible macromolecular damage. ELISA confirmed markedly elevated levels of malondialdehyde (MDA), an end-product of lipid peroxidation, in Fe/Zn-CNZ@LOx@PEG-treated cells (Fig. 5D). In contrast, superoxide dismutase (SOD) activity—a primary antioxidant enzyme—was significantly down-regulated (Fig. 5E). This inverse SOD/MDA relationship reveals a dual damage mechanism: excessive superoxide (O_2_·^−^) overwhelms SOD scavenging capacity, triggering lipid peroxidation chain reactions that culminate in plasma membrane integrity loss.

Dynamic monitoring using dihydroethidium (DHE) probes further corroborated specific intracellular superoxide surge induction by Fe/Zn-CNZ@LOx@PEG (Fig. 5F and G). The ~40-fold fluorescence enhancement from ethidium-–DNA adducts generated by O_2_·^−^ oxidation provides direct evidence for the Zn-enhanced Fenton-driven oxidative storm. Collectively, Fe/Zn-CNZ@LOx@PEG establishes a self-reinforcing oxidative stress microenvironment via a tiered cascade: “catalytic activity amplification → antioxidant system inhibition → biomolecular damage”.

### Fe/Zn-CNZ@LOx@PEG triggers ferroptosis and energy metabolism dysregulation in PCa cells

Ferroptosis is an iron-dependent, LPO-driven programmed cell death regulated by intracellular antioxidant systems. GSH serves as an essential cofactor for glutathione peroxidase 4 (GPX4), reducing phospholipid hydroperoxides (PLOOH) to inhibit LPO accumulation [28,29]. GPX4 inactivation is a pivotal ferroptosis trigger, as its activity critically depends on GSH supply—itself reliant on cystine uptake via the membrane transporter SLC7A11 (core subunit of system Xc^–^). SLC7A11 inhibition causes GSH depletion and consequent GPX4 inactivation [30]. Additionally, repressed expression or function of the iron exporter FPN1 exacerbates intracellular iron accumulation, amplifying ROS generation and LPO via the Fenton reaction, ultimately causing irreversible plasma membrane failure [31].

**Fig. 6.**
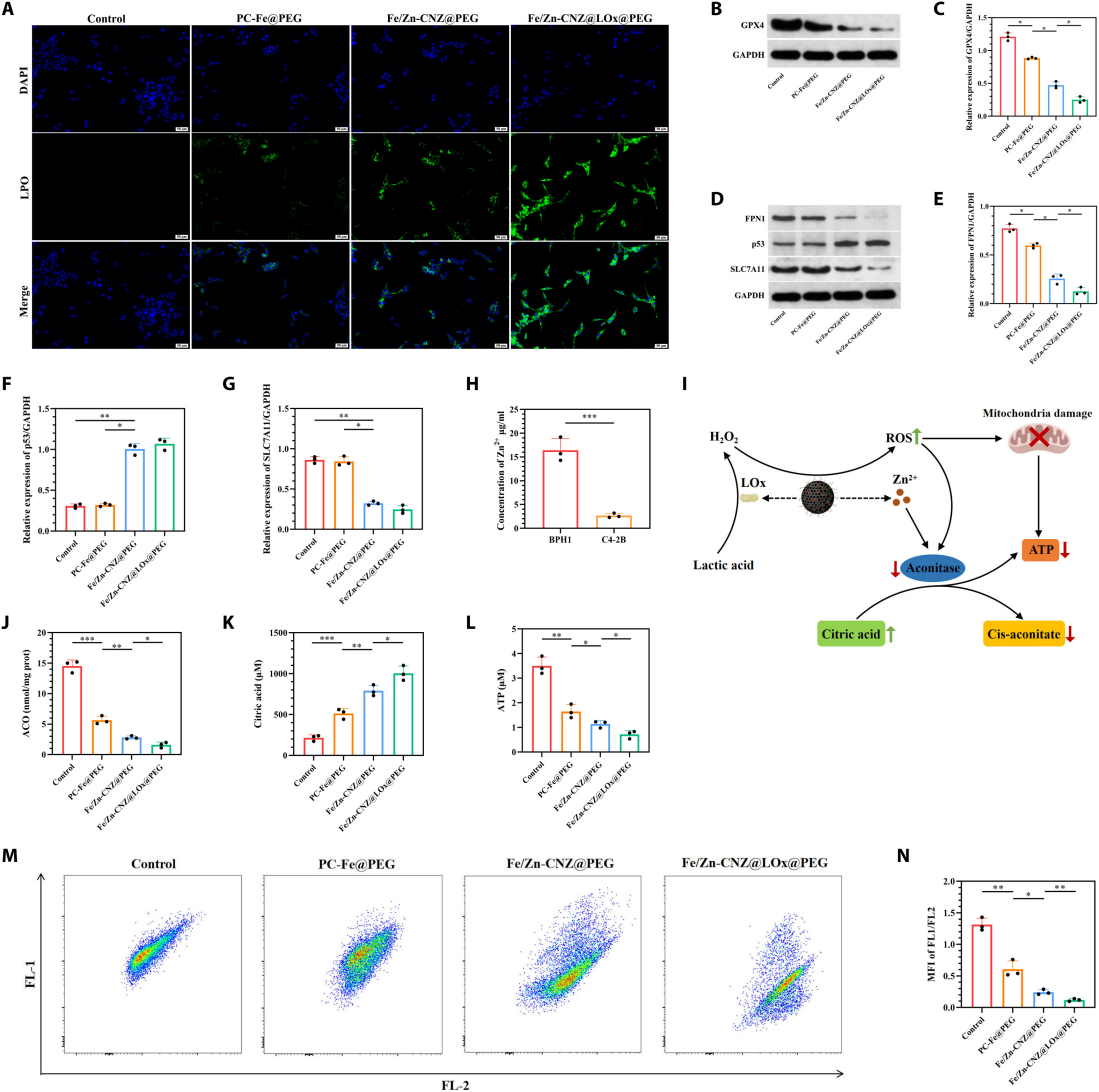
Ferroptosis induction by Fe/Zn-CNZ@LOx@PEG in PCa cells. (A) Lipid peroxidation (LPO) detection in MyC-CaP cells via C11-BODIPY^581/591^ probe (scale bar, 50 μm; *n* = 3). (B) Western blot analysis of GPX4 protein expression. (C) Quantitative analysis of GPX4 expression. (D) Western blot analysis of FPN1, p53, and SLC7A11 protein expression. (E to G) Quantitative analysis of FPN1, p53, and SLC7A11 expression. (H) Zinc ion concentration in BPH1 and C4-2B cells (*n* = 3). (I) Schematic illustration of tumor cell energy metabolism regulation by Fe/Zn-CNZ@LOx@PEG. (J) Aconitase activity measurements (*n* = 3). (K) Citric acid content analysis (*n* = 3). (L) ATP levels in different groups (*n* = 3). (M) Flow cytometric (FCM) analysis of MMP using JC-1 probe (*n* = 3) and (N) corresponding quantitative data. *P < 0.05, **P < 0.01, ***P < 0.001.

Using the C11-BODIPY^581/591^ fluorescent probe detecting ferroptosis-characteristic LPO (red fluorescence when reduced, green fluorescence upon oxidation; red/green ratio directly reporting LPO levels), we observed that all 3 materials—PC-Fe@PEG, Fe/Zn-CNZ@PEG, and Fe/Zn-CNZ@LOx@PEG—exhibited significant green fluorescence, with Fe/Zn-CNZ@LOx@PEG demonstrating the highest fluorescence intensity, indicating superior ROS-generating capability (Fig. 6A). Western blotting confirmed that Fe/Zn-CNZ@LOx@PEG significantly suppressed GPX4 expression in PCa cells, exceeding PC-Fe@PEG and Fe/Zn-CNZ@PEG effects (Fig. 6B and C), establishing that Fe/Zn-CNZ@LOx@PEG promotes ferroptosis by targeting GPX4. To further elucidate the ferroptosis-inducing effect of Fe/Zn-CNZ@LOx@PEG on tumor cells, PCa cells were pretreated with the ferroptosis inhibitor Ferrostatin-1 (Fer-1), the apoptosis inhibitor Z-VAD-FMK (ZVF), and the necroptosis inhibitor Necrostatin-1 (Nec-1), respectively. As shown in Figs. S6 to S8, Fer-1 significantly rescued the decrease in cell viability (Fig. S6) and suppressed LPO accumulation induced by Fe/Zn-CNZ@LOx@PEG (Fig. S7), but it did not reverse the depletion of intracellular GSH (Fig. S8). These results indicate that while Fer-1 exerts its protective effect downstream in the ferroptosis pathway by scavenging lipid radicals and inhibiting the propagation of lipid peroxidation, it does not directly replenish GSH, enhance GPx4 activity, or eliminate radicals. Thus, Fer-1 cannot counteract the GSH depletion caused by the nanoparticles. In contrast, ZVF and Nec-1 only partially attenuated cell death without inhibiting ferroptosis. Collectively, these findings confirm that Fe/Zn-CNZ@LOx@PEG induces tumor cell death primarily through ferroptosis, which is mediated by GSH depletion.

Critically, Zn^2+^ plays a key regulatory role in ferroptosis. Evidence indicates that elevated Zn^2+^ stabilizes p53 protein and enhances its transcriptional activity. Activated p53 directly binds the SLC7A11 gene promoter, inhibiting its expression [32,33]. Down-regulation of SLC7A11—the core subunit of the cystine/glutamate antiporter (system Xc^–^)—leads to cysteine depletion, hindering GSH biosynthesis [30,34]. GSH deficiency impairs GPX4 activity (dependent on GSH as a cofactor), crippling cellular ROS-scavenging and LPO-suppression capacity, ultimately triggering runaway iron-dependent LPO and ferroptosis [30,34]. Our study confirmed that Fe/Zn-CNZ@LOx@PEG significantly down-regulated FPN1 and SLC7A11 expression (Fig. 6D, E, and G) while up-regulating p53 levels (Fig. 6D to F), validating the core function of the Zn^2+^/p53/SLC7A11 axis in Fe/Zn-CNZ@LOx@PEG-induced ferroptosis. It is important to note that the present study primarily demonstrates correlative changes in the key players of this pathway. While our results are consistent with the established model, they do not provide direct causal evidence for Zn^2+^-mediated p53 activation, leading to transcriptional repression of SLC7A11 in our specific system. As rightly suggested, future investigations employing genetic approaches such as p53-knockout models or SLC7A11 promoter-reporter assays would be required to definitively establish the direct transcriptional regulation and the functional causality within this axis in the context of our nanoparticle treatment. Nonetheless, the concordance of our protein expression data with this major ferroptosis pathway strongly implicates its contribution to the mechanism of action.

Intracellular zinc dyshomeostasis is a hallmark of PCa progression. Unlike normal prostate epithelial cells that maintain high physiological zinc levels to support functions like citrate synthesis, PCa cells exhibit a hypozinc microenvironment due to dysfunctional ZIP family importer activity and enhanced zinc efflux [35,36]. This low zinc state signifies the TME’s pathological nature and promotes malignancy by activating invasion-related signaling pathways [e.g., phosphatidylinositol 3-kinase (PI3K)/AKT and nuclear factor κB (NF-κB)] and impairing zinc-dependent tumor suppressors (e.g., p53 and E-cadherin) [35,36]. Our data confirmed significantly lower zinc levels in human PCa C4-2B cells compared to normal prostate BPH1 cells (Fig. 6H), supporting this conclusion. Therefore, controlled elevation of intracellular zinc may represent a novel anticancer strategy.

Furthermore, Zn^2+^ plays a pivotal role in tumor metabolic reprogramming by targeting mitochondrial enzymes, particularly aconitase [37]. High Zn^2+^ concentrations specifically disrupt the [4Fe-4S] cluster active site of aconitase, leading to Fe–S cluster disassembly and enzyme inhibition, which blocks the conversion of citrate to cis-aconitate in the TCA cycle [17,37,38]. Concomitant energy metabolism dysfunction significantly reduces ATP synthesis efficiency (Fig. 6I), forcing tumor cells to rely on Warburg glycolysis for energy production and shunting intermediates toward biosynthetic pathways, ultimately driving adaptive metabolic reprogramming.

Our study further revealed Fe/Zn-CNZ@LOx@PEG’s metabolic impact: significant aconitase activity down-regulation (Fig. 6J), increased citrate content (Fig. 6K), and diminished ATP generation (Fig. 6L) upon treatment, demonstrating a Zn^2+^-mediated blockade of a key TCA cycle step and consequent energy stress. Crucially, this TCA cycle disruption and the associated energy crisis are not merely bystander effects but are posited to directly sensitize PCa cells to ferroptosis through multiple converging mechanisms. First, TCA cycle dysfunction can limit the generation of reducing equivalents, such as NADPH (reduced form of nicotinamide adenine dinucleotide phosphate), which is essential for regenerating GSH and sustaining the activity of GPX4—the central guardian against ferroptosis. NADPH limitation thus exacerbates the GSH depletion initiated by the nanoplatform, creating a vicious cycle that cripples the cellular antioxidant defense. Second, the metabolic rewiring and potential alterations in acetyl-coenzyme A flux resulting from citrate accumulation and ATP depletion may reprogram lipid metabolism, potentially increasing the pool of polyunsaturated fatty acids (PUFAs) that are susceptible to peroxidation—the fundamental biochemical driver of ferroptosis. The profound MMP collapse observed (Fig. 6M and N) further signifies severe organelle dysfunction that amplifies oxidative stress. Collectively, these alterations establish a feed-forward loop where Zn^2+^-induced metabolic perturbation (aconitase inhibition → TCA cycle disruption → energy/redox stress) synergizes with the nanoparticle’s catalytic ROS generation to dramatically lower the threshold for ferroptosis execution. This elucidated link between targeted metabolic interference and ferroptosis susceptibility underscores the novelty of our “metabolic-immuno” co-regulation strategy, which disrupts tumor cell vitality not only by direct catalytic attack but also by reprogramming core metabolic pathways to create a cell-intrinsic vulnerability to iron-dependent death.

It is noteworthy that the robust antitumor immune activation observed in this study may not solely originate from the indirect pathway of Zn^2+^-mediated ferroptosis and ICD in tumor cells. Accumulating evidence suggests that Zn^2+^ itself, acting as a crucial intracellular second messenger (a process termed “zinc signaling”), can directly modulate the functional state of immune cells within the TME [39]. For instance, in T cells, Zn^2+^ is indispensable for T cell receptor signaling, activation, and the differentiation of effector functions; zinc deficiency is associated with reduced numbers and impaired function of both CD4^+^ and CD8^+^ T cells [40]. Similarly, in DCs, sufficient zinc levels are essential for effective DC maturation and antigen-presenting capacity [41], which aligns well with our experimental observation of up-regulated CD80/CD86 expression. Therefore, the Zn^2+^ released from the Fe/Zn-CNZ@LOx@PEG platform likely plays a dual role in the TME. On one hand, it corrects zinc deficiency in tumor cells, disrupting their metabolic homeostasis and inducing ICD, thereby releasing tumor antigens and danger signals. On the other hand, it may directly act on infiltrating immune cells such as T cells and DCs, potentiating their activation, proliferation, and cytotoxic functions. Together, these actions form a synergistic “metabolism-immunity” positive regulatory circuit. Although directly disentangling the indirect (via tumor cells) from the direct (on immune cells) effects of Zn^2+^ poses a technical challenge within the model system used in this work, this dual-action mechanism provides a more integrated theoretical framework to explain the potent systemic antitumor immune response and the marked synergistic effect with αPD-1 therapy observed in our study.

### Fe/Zn-CNZ@LOx@PEG induces ICD

**Fig. 7.**
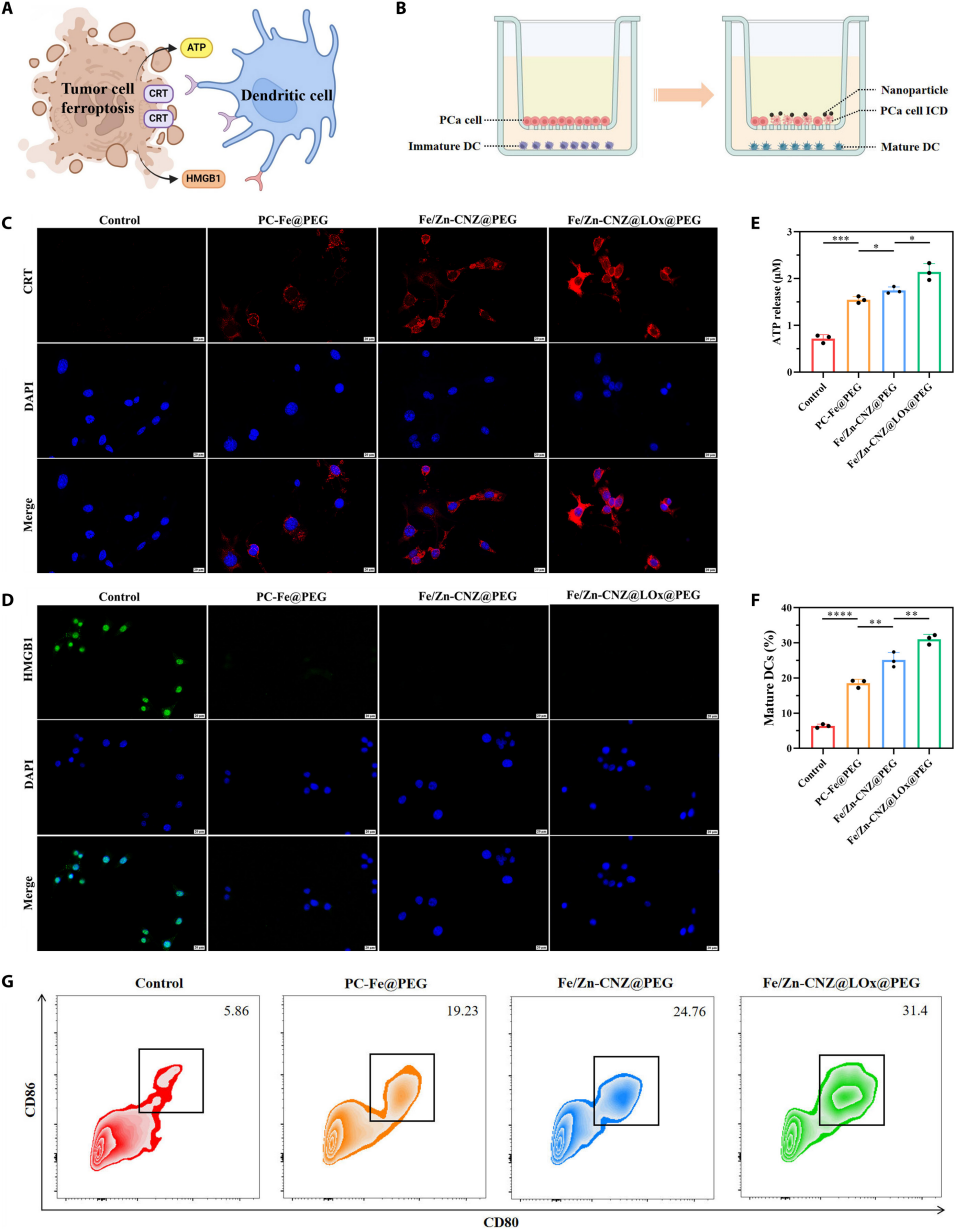
ICD induction by Fe/Zn-CNZ@LOx@PEG in PCa cells. (A) Mechanistic diagram of Fe/Zn-CNZ@LOx@PEG-induced ICD. (B) Schematic workflow for in vitro DC maturation assessment. (C) Fluorescent imaging of CRT and (D) HMGB1 expression in MyC-CaP cells (scale bar, 20 μm). (E) Extracellular ATP levels measured via ATP Assay Kit (*n* = 3). (G) FCM analysis of DC maturation induced by treated MyC-CaP cells (*n* = 3) and (F) corresponding quantitative data. *P < 0.05, **P < 0.01, ***P < 0.001, ****P < 0.0001.

ICD is a specific form of programmed cell death characterized by the release of TAAs and DAMPs following membrane disintegration [42]. This activates host antitumor immunity through coordinated signaling (Fig. 7A): CRT translocates to the cell surface during endoplasmic reticulum stress, acting as an “eat-me” signal recognized by DCs to enhance phagocytosis [42]. High mobility group box 1 (HMGB1) is released extracellularly upon nuclear or mitochondrial membrane damage, driving pro-inflammatory cytokine secretion and immune cell recruitment via Toll-like receptor (TLR)/receptor for advanced glycation endproducts (RAGE) activation [43–45]. Extracellular ATP enhances DC chemoattraction to the TME via P2X7 receptor signaling, creating an immune-activating feedback loop. These cascading events ultimately present tumor antigens to T cells, initiating antigen-specific immune responses [44,45].

The Transwell chamber was used to detect the effect of Fe/Zn-CNZ@LOx@PEG on the ICD of PCa cells (Fig. 7B). Our study found that ferroptosis induced by Fe/Zn-CNZ@LOx@PEG strongly correlated with ICD mechanisms. Immunofluorescence analysis showed significantly up-regulated surface CRT expression in Fe/Zn-CNZ@LOx@PEG-treated cells (Fig. 7C), with fluorescence intensity exceeding control, PC-Fe@PEG, and Fe/Zn-CNZ@PEG groups, indicating enhanced “eat-me” signal exposure. Simultaneously, loss of nuclear HMGB1 immunofluorescence signal with significantly elevated extracellular HMGB1 levels (Fig. 7D) suggested protein release through mitochondrial/nuclear rupture, activating inflammatory pathways. Furthermore, extracellular ATP levels showed a dose-dependent increase post-treatment (Fig. 7E), correlating positively with enhanced DC chemotaxis, confirming ATP’s critical role as a “danger signal” for immune cell recruitment to the TME.

DC activation analysis using flow cytometry of Transwell cocultures confirmed Fe/Zn-CNZ@LOx@PEG’s immunomodulatory capacity. Following coculture with Fe/Zn-CNZ@LOx@PEGG-treated cells, immature DCs (imDCs) exhibited significantly elevated expression of costimulatory molecules CD80 and CD86 compared to PC-Fe@PEG or Fe/Zn-CNZ@PEG exposure (Fig. 7F and G). This demonstrates that Fe/Zn-CNZ@LOx@PEG not only induces ferroptosis-related ICD but also directly potentiates DC antigen presentation, driving their maturation. Mature DCs present TAAs to CD8^+^ T cells via major histocompatibility complex class I (MHC-I) and secrete cytokines (e.g., TNF-α and IFN-γ), promoting T cell differentiation into effector T cells (Teff) for tumor-specific killing [46,47].

Notably, Fe/Zn-CNZ@LOx@PEG’s immunomodulation stems from its multi-target synergy. Zn^2+^ stabilizes p53 and inhibits SLC7A11 expression, causing GSH depletion/GPX4 inactivation and ferroptosis. Simultaneously, Zn^2+^ inhibits mitochondrial aconitase, blocking the TCA cycle and forcing Warburg metabolism, exacerbating oxidative stress and mitochondrial dysfunction—providing the metabolic foundation for ICD [35,38,48]. Furthermore, Fe/Zn-CNZ@LOx@PEG catalyzes ROS generation via the Fenton reaction, promoting LPO-induced ferroptosis and oxidizing HMGB1/CRT to enhance their immunostimulatory activity. This “Ferroptosis-ICD-Immune Activation” cascade provides a novel theoretical basis for overcoming PCa immune tolerance.

In summary, Fe/Zn-CNZ@LOx@PEG triggers ICD via ferroptosis, activating innate immunity (via CRT/HMGB1/ATP release) and adaptive immunity (via DC maturation/antigen presentation). This dual immunomodulatory strategy offers new therapeutic targets, particularly for PCa with limited responses to ICIs.

### Fe/Zn-CNZ@LOx@PEG demonstrates excellent biocompatibility

**Fig. 8.**
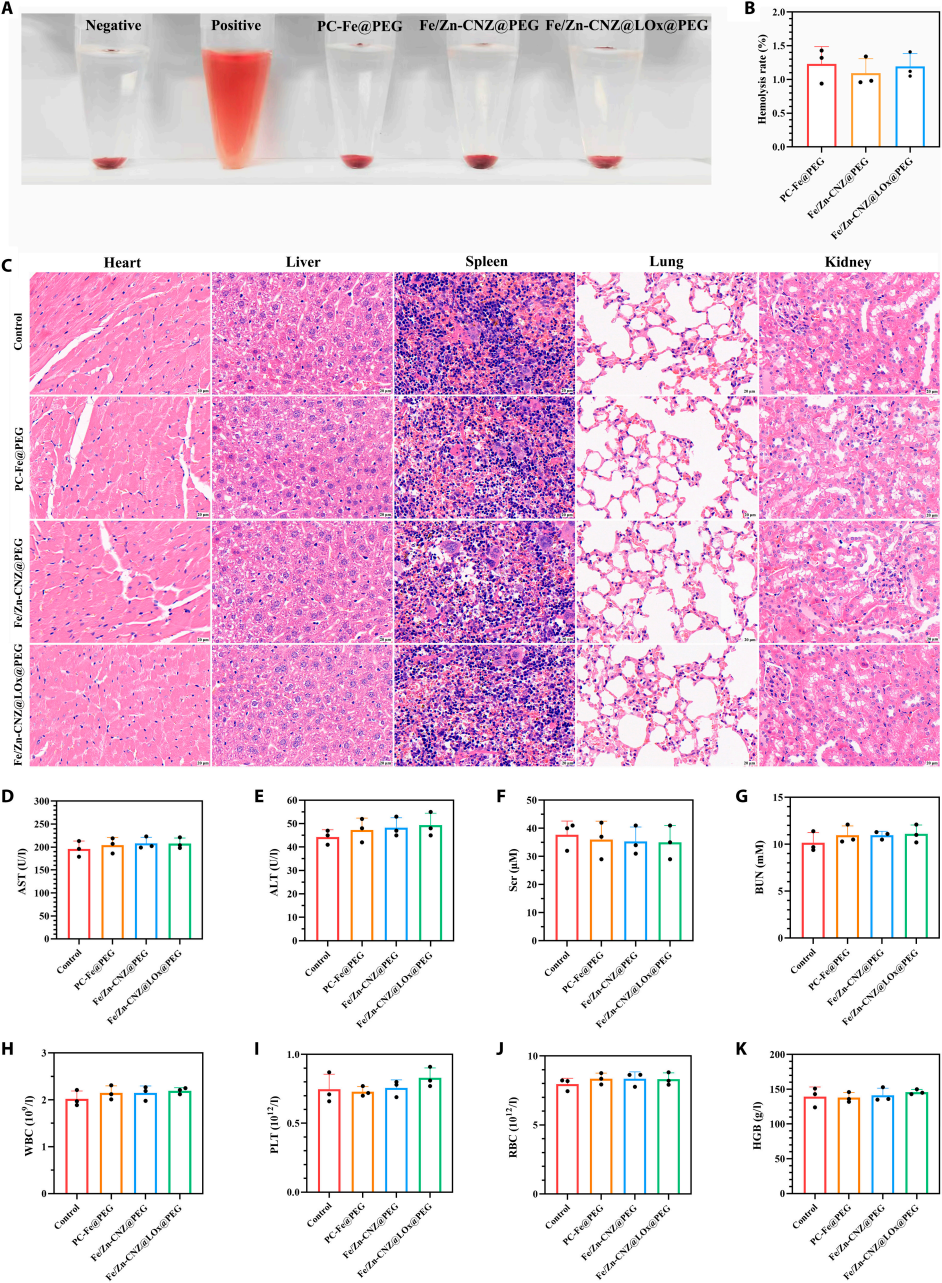
Biocompatibility evaluation of Fe/Zn-CNZ@LOx@PEG. (A) Blood compatibility assay of Fe/Zn-CNZ@LOx@PEG and (B) corresponding quantitative analysis. (C) H&E staining of major organs (heart, liver, spleen, lung, kidney). Scale bar, 20 μm. (D) Serum aspartate aminotransferase (AST), (E) alanine aminotransferase (ALT), (F) serum creatinine (Scr), and (G) blood urea nitrogen (BUN) levels in mice (*n* = 3). (H) White blood cell (WBC), (I) platelet (PLT), (J) red blood cell (RBC), and (K) hemoglobin (HGB) counts in serum (*n* = 3).

Biocompatibility is paramount for the clinical translation of novel nanotherapeutics. We systematically evaluated the hemocompatibility and systemic toxicity of PC-Fe@PEG, Fe/Zn-CNZ@PEG, and Fe/Zn-CNZ@LOx@PEG to validate their potential as PCa-targeted delivery vectors. Hemolysis assays showed that both nanomaterials induced minimal erythrocyte membrane damage (hemolysis rate <5%; Fig. 8A and B), confirming excellent blood compatibility and adherence to international standards for biomedical materials, supporting further in vivo use.

Intravenous injection revealed no acute toxicity: No weight loss, behavioral abnormalities, or mortality occurred over 7 d post-injection. Histopathological H&E staining of major organs (heart, liver, spleen, lungs, kidneys) showed no significant inflammation, necrosis, or fibrosis (Fig. 8C), indicating no structural damage at the tested dose.

Serum biochemistry (ALT, AST, BUN, Cr; Fig. 8D to G) remained within normal physiological ranges, indicating unaffected hepatic and renal function. CBCs (WBC, PLT, RBC, HGB) showed no significant alterations (Fig. 8H to K), confirming no adverse effects on hematopoiesis. These multi-faceted toxicity data support the low systemic toxicity profile of Fe/Zn-CNZ@LOx@PEG.

The exceptional biocompatibility likely stems from PEG surface modification. PEG creates a steric shield that minimizes nonspecific biomolecular interactions, reducing macrophage uptake and complement activation, thereby lowering immunogenicity. Moreover, controlled Zn^2+^ release mitigates risks of free metal ion toxicity, while the stable PC-Fe framework ensures slow metal metabolism, preventing chronic metal accumulation effects. This design strategy achieves targeted efficacy while avoiding common toxicity pitfalls of metal-based nanomaterials.

Collectively, Fe/Zn-CNZ@LOx@PEG exhibits excellent biocompatibility in vitro and in vivo, with a toxicity profile comparable to established biodegradable nanomaterials. This validates its preclinical safety and lays the groundwork for future studies on long-term biodistribution and organ clearance—a crucial step toward clinical translation.

### Fe/Zn-CNZ@LOx@PEG’s in vivo antitumor efficacy

Despite the breakthrough of ICIs targeting the PD-1/PD-L1 axis, their efficacy in PCa remains limited by low response rates (~20%) and immune tolerance mechanisms (e.g., T cell exhaustion). To overcome this, we propose combining Fe/Zn-CNZ@LOx@PEG-induced ferroptosis/ICD with αPD-1 therapy. This strategy leverages ferroptosis to expose TAAs and activate DCs, aiming to break immune tolerance and enhance αPD-1 efficacy by remodeling the TME.

**Fig. 9.**
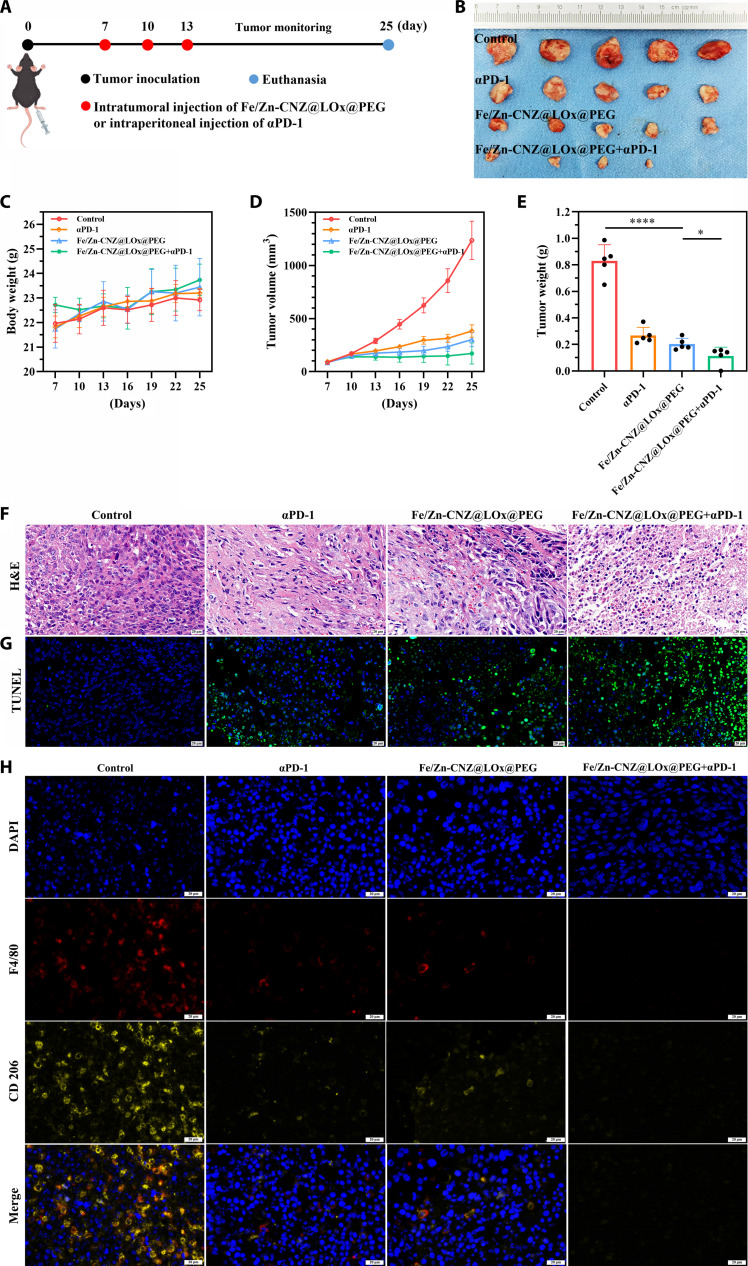
In vivo antitumor efficacy of Fe/Zn-CNZ@LOx@PEG combined with αPD-1. (A) Schematic illustration of nanomaterial and αPD-1 administration in tumor-bearing mice. (B) Macroscopic images of excised tumors (*n* = 5). (C) Body weight dynamics across treatment groups (*n* = 5). (D) Tumor volume progression over time (*n* = 5). (E) Final tumor weights (*n* = 5). (F) H&E and (G) TUNEL staining of tumor tissues post-treatment (scale bar, 50 μm). (H) M2 macrophage staining of tumor tissues post-treatment (scale bar, 20 μm). *P < 0.05, ****P < 0.0001.

In subcutaneous MyC-CaP tumors (C57BL/6 mice, *n* = 5 per group), Fe/Zn-CNZ@LOx@PEG combined with αPD-1 (intraperitoneally) yielded superior antitumor effects compared to either monotherapy (PBS, αPD-1, or Fe/Zn-CNZ@LOx@PEG alone) by day 25 (Fig. 9A, C, and D). Tumor photographs (Fig. 9B) and weight measurements (Fig. 9E) confirmed maximal tumor mass reduction and extensive necrosis in the combination group.

Histopathology revealed the synergy: H&E staining showed focal necrosis with Fe/Zn-CNZ@LOx@PEG alone versus widespread cellular disintegration, pyknosis, and interstitial hemorrhage in the combination group (Fig. 9F), consistent with ferroptosis-induced membrane damage and ICD pathology. TUNEL (terminal deoxynucleotidyl transferase–mediated deoxyuridine triphosphate nick end labeling) staining confirmed significantly higher apoptotic cell death in the combination group (Fig. 9G), indicating that ferroptotic death releases DAMPs activating DCs, thus amplifying αPD-1-driven T cell responses. In addition, tumor-associated macrophages (M2 macrophages) were examined. The results showed that the combined therapy of Fe/Zn-CNZ@LOx@PEG and αPD-1 could significantly inhibit the infiltration of M2 macrophages in the tumor tissue (Fig. 9H).

Mechanistically, Fe/Zn-CNZ@LOx@PEG enables multi-targeted antitumor effects: Zn^2+^ disrupts cellular energy metabolism by inhibiting the TCA cycle enzyme aconitase, thereby forcing a Warburg glycolytic shift that elevates ROS production. Simultaneously, LOx catalyzes lactate oxidation to generate abundant H_2_O_2_, which intensifies the Fe^2+^-mediated Fenton reaction, leading to amplified ROS burst and LPO—directly inducing ferroptosis. Additionally, Zn^2+^ further promotes ferroptosis through p53-mediated down-regulation of SLC7A11 and depletion of GSH. Together, these actions form a coordinated “metabolism–oxidation–immunity” regulatory triad, establishing a biochemical basis for enhanced responsiveness to ICIs. In conclusion, Fe/Zn-CNZ@LOx@PEG enhances αPD-1 efficacy by inducing ferroptosis, exposing TAAs, and remodeling metabolic homeostasis. This validates the ferroptosis-immunotherapy synergy and provides an innovative strategy to overcome immune resistance in PCa.

It should be noted that a detailed investigation into the pharmacokinetic profile, biodistribution, and passive/active tumor-targeting efficiency of Fe/Zn-CNZ@LOx@PEG, while beyond the scope of this foundational study, is essential for clinical translation and will be a focus of our subsequent research.

### Fe/Zn-CNZ@LOx@PEG and αPD-1 synergy suppresses distant tumors

**Fig. 10.**
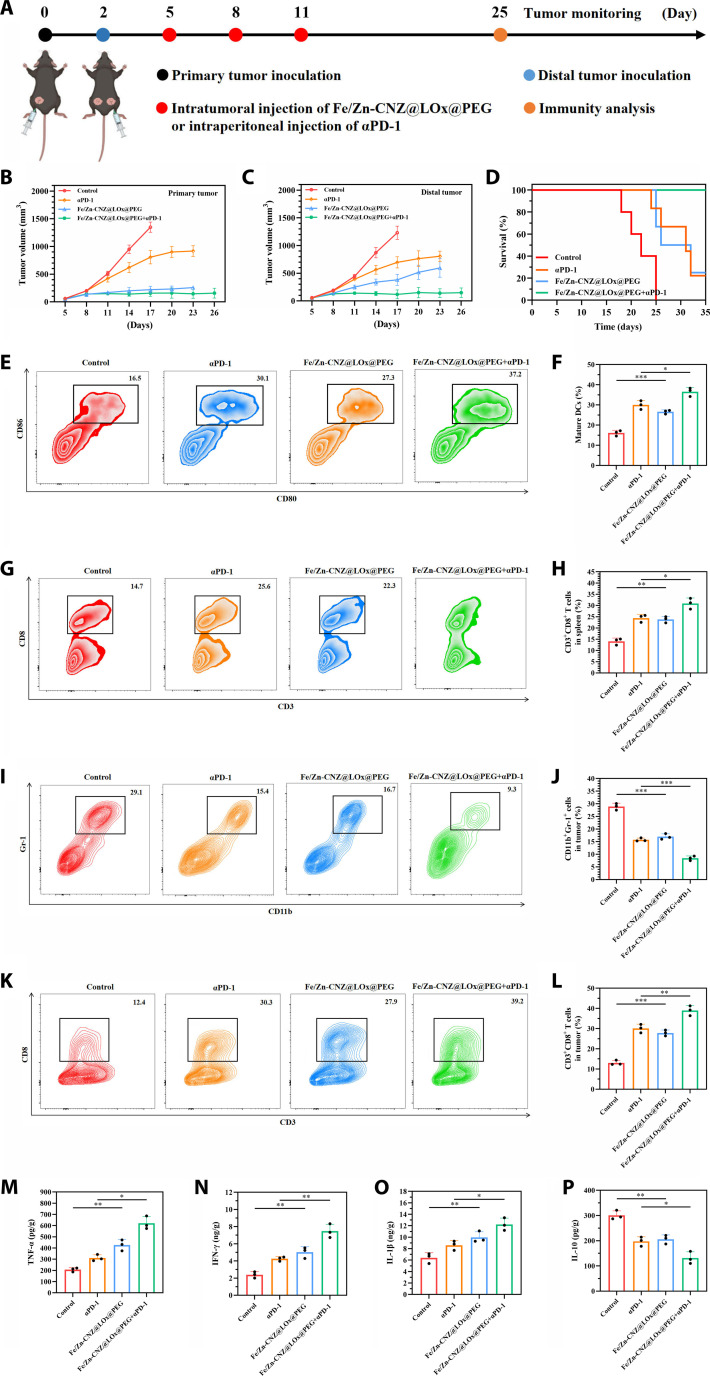
Systemic antitumor immunity and immune profiling after Fe/Zn-CNZ@LOx@PEG + αPD-1 treatment. (A) Schematic of bilateral subcutaneous tumor model treatment protocol. (B) Primary tumor growth curves (*n* = 5). (C) Distant tumor volume progression (*n* = 5). (D) Survival analysis of treated mice (*n* = 5). (E) FCM analysis of mature DCs in tumor-draining LNs (*n* = 3) and (F) corresponding quantitative analysis. (G) CD8^+^ T cell proportions in spleens (*n* = 3) and (H) corresponding quantitative analysis. (I) MDSC proportions in tumors (*n* = 3) and (J) corresponding quantitative analysis. (K) CD8^+^ T cell infiltration in tumors (*n* = 3) and (L) corresponding quantitative analysis. Cytokine profiling of (M) TNF-α, (N) IFN-γ, (O) IL-1β, and (P) IL-10 in tumor tissues via ELISA (*n* = 3). *P < 0.05, **P < 0.01, ***P < 0.001.

To evaluate systemic antitumor effects, we established bilateral MyC-CaP subcutaneous tumors. The intratumoral injection of Fe/Zn-CNZ@LOx@PEG plus αPD-1 (intraperitoneally) achieved significantly superior growth inhibition of both primary and distant tumors compared to monotherapies (Fig. 10A to C). Critically, combination therapy markedly enhanced survival, with all combination-treated mice surviving the 35-d study, while others succumbed to tumor burden (Fig. 10D).

Flow cytometry revealed the immunological basis: Combination therapy significantly increased mature DCs (CD80^+^ CD86^+^) in tumor-draining LNs (Fig. 10E and F) and boosted splenic CD8^+^ T cell frequencies (Fig. 10G and H), reflecting enhanced antigen presentation and systemic T cell activation. Distant tumors exhibited significantly reduced MDSCs (CD11b^+^ Gr1^+^; Fig. 10I and J) and increased CD8^+^ T cell infiltration (Fig. 10K and L), demonstrating successful reversal of the distal immunosuppressive TME.

Cytokine profiling confirmed a potentiated pro-inflammatory milieu: Combination therapy significantly elevated TNF-α, IFN-γ, and IL-1β while suppressing IL-10 levels within primary tumors (Fig. 10M to P), establishing a TME favoring antitumor immunity.

In summary, combination therapy orchestrates multi-mechanistic synergy: Fe/Zn-CNZ@LOx@PEG induces ferroptosis/ICD, releasing TAAs and activating DCs; αPD-1 blocks PD-1/PD-L1, reversing T cell exhaustion. This remodels the TME (promoting Teff infiltration, suppressing MDSCs), enhances pro-inflammatory signaling, and establishes systemic antitumor immunity effective against both local and distant disease, offering significant clinical potential.

## Conclusion

In conclusion, this study successfully developed an intelligent, TME-responsive nanoreactor, Fe/Zn-CNZ@LOx@PEG, which establishes a novel “metabolic-immuno” co-regulation paradigm for the synergistic ferroptosis-immunotherapy of PCa. The core innovation lies in the spatiotemporal integration of 3 key therapeutic modalities: (a) the restoration of zinc homeostasis to reverse a hallmark metabolic deficiency in PCa, (b) a zinc-augmented bimetallic (Fe–Zn) nanocatalytic system for self-amplifying ROS generation via lactate-fueled cascade reactions, and (c) the consequent induction of ICD. This nanoplatform concurrently addresses major therapeutic gaps in PCa, including the singularity of molecular targeting, the low response rates of monotherapies like ICIs, and the biological inertia of conventional nanocarriers. Mechanistically, Fe/Zn-CNZ@LOx@PEG triggers profound ferroptosis through a multi-pronged attack: catalytic GSH depletion, Zn^2+^/p53-mediated repression of SLC7A11, and disruption of mitochondrial metabolism via aconitase inhibition. The resultant ferroptosis acts as a potent ICD inducer, releasing DAMPs and TAAs that promote DC maturation, enhance cytotoxic T lymphocyte infiltration, and reprogram the immunosuppressive TME. The compelling in vivo efficacy, demonstrated by significant suppression of both local and distant tumors in a bilateral model and a strong synergy with αPD-1 therapy, coupled with excellent biocompatibility, underscores the high translational potential of this strategy.

Despite these promising results, several limitations of the present study warrant acknowledgment and present clear avenues for future research. First, while our data strongly implicate the Zn^2+^/p53/SLC7A11 axis, direct causal evidence for Zn^2+^-mediated p53 activation leading to transcriptional repression of SLC7A11 in this specific context requires validation using genetic tools such as p53-knockout models or SLC7A11 promoter-reporter assays. Second, the proposed molecular mechanism of Zn^2+^ in enhancing Fenton catalysis by stabilizing high-valent iron intermediates, though consistent with literature on bimetallic systems, would benefit from more direct spectroscopic evidence (e.g., in situ Mossbauer or x-ray absorption spectroscopy) to conclusively characterize the transient catalytic species. Third, a systematic investigation into the pharmacokinetic profile, biodistribution, and passive/active tumor-targeting efficiency of Fe/Zn-CNZ@LOx@PEG is essential for clinical translation and will be a critical focus of subsequent studies. Fourth, the therapeutic efficacy was evaluated in immunocompetent but subcutaneous tumor models; future work should employ more clinically relevant models, such as orthotopic or spontaneous PCa models, to better assess the impact on the native prostate TME and metastatic progression. Finally, long-term biosafety studies monitoring potential metal ion accumulation and organ clearance over extended periods are necessary to fully de-risk this metal-based nanoplatform.

Looking forward, this work opens several exciting research directions. The modular design of the nanoreactor allows for the potential integration of other therapeutic agents (e.g., chemotherapeutics and other immunomodulators) or targeting ligands to further enhance specificity and efficacy. Exploring combinations with other emerging immunotherapies beyond PD-1 blockade could help overcome diverse resistance mechanisms. Furthermore, the “metabolic-immuno” co-regulation principle demonstrated here could be adapted to target other cancer types characterized by similar metabolic dysregulations. In summary, Fe/Zn-CNZ@LOx@PEG represents a robust and innovative nanomedicine strategy that effectively bridges catalytic therapy, metabolic reprogramming, and immunotherapy, offering a compelling blueprint for next-generation combination therapies against advanced and treatment-resistant cancers.

## Data Availability

Data will be made available on request.
