# Supplementary material for: Restoring Zinc Homeostasis via a Bimetallic Nanozyme to Amplify Ferroptosis and Antitumor Immunity for Prostate Cancer Treatment
Source: Biomater Res. 2026 Jun 26;30:0378. doi: 10.34133/bmr.0378 (PMC13305028; doi:10.34133/bmr.0378)
Supplement: Supplementary 1 — Figs. S1 to S8 [file bmr.0378.f1.docx]

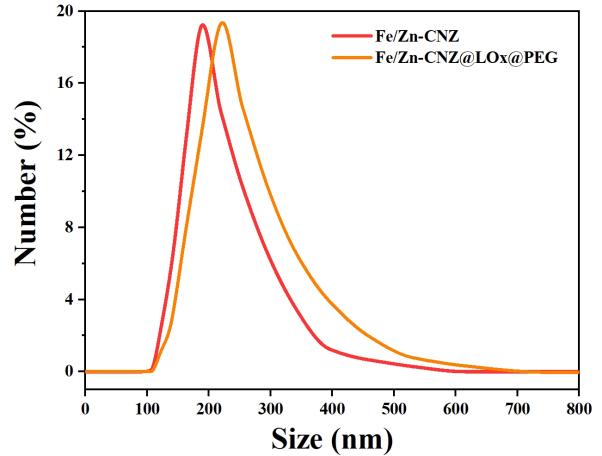


**Figure S1.** Dynamic light scattering (DLS) analysis of Fe/Zn-CNZ and Fe/Zn-CNZ@LOx@PEG.


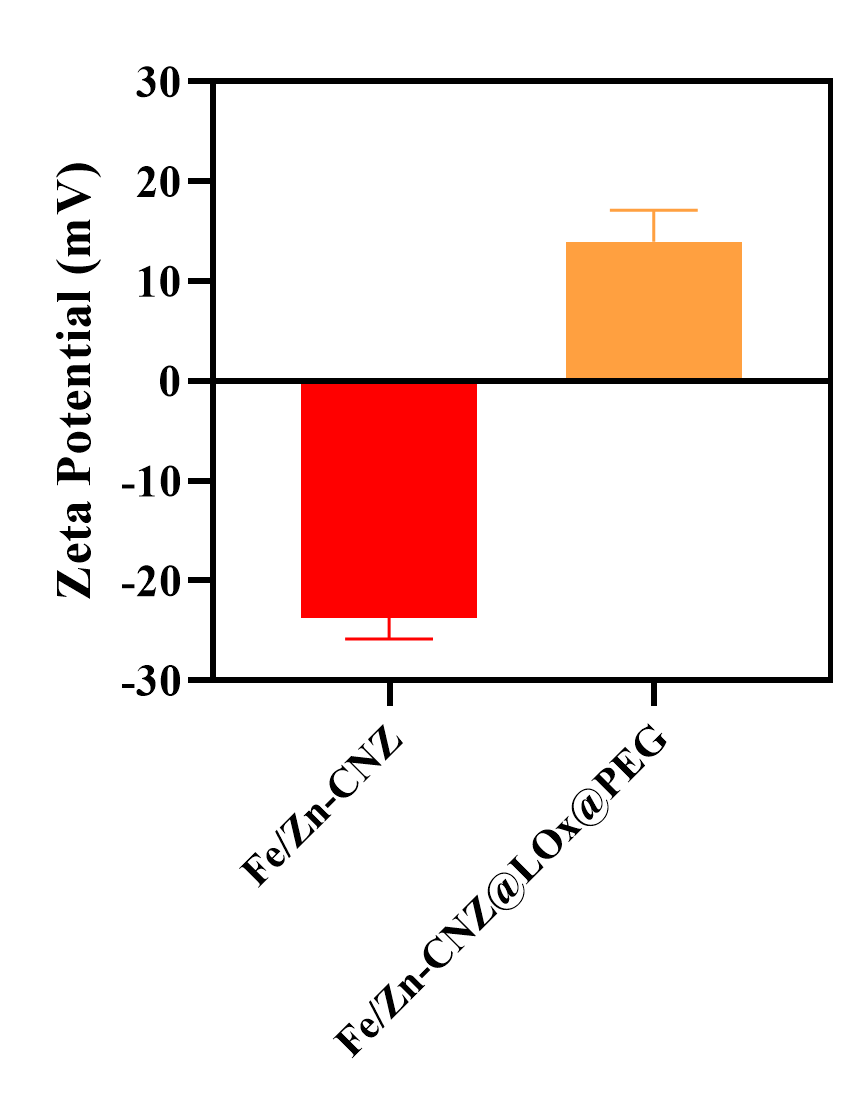


**Figure S2.** Zeta potential measurements of Fe/Zn-CNZ and Fe/Zn-CNZ@LOx@PEG.


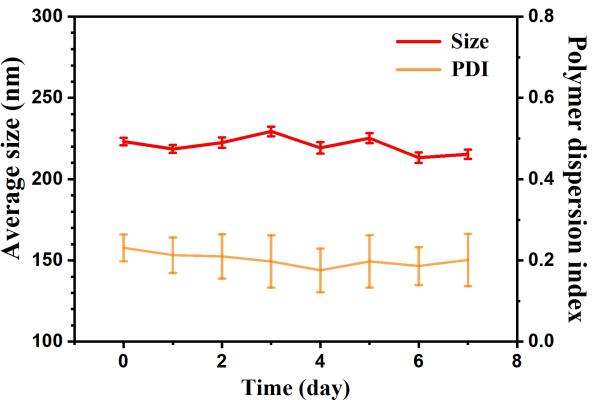


**Figure S3.** The dispersion index of Fe/Zn-CNZ@LOx@PEG.


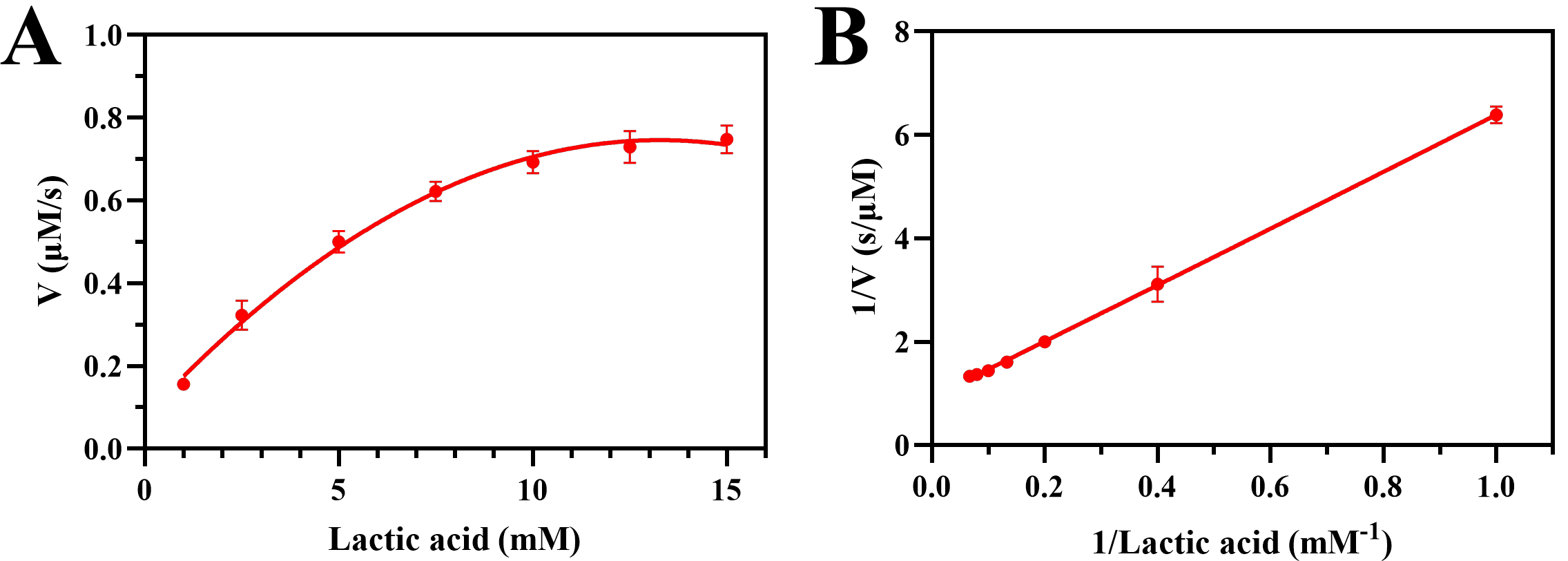


**Figure S4.** (A) Michaelis–Menten kinetic analysis and (B) Lineweaver–Burk plot for Fe/Zn-CNZ@LOx@PEG with lactic acid as substrate.


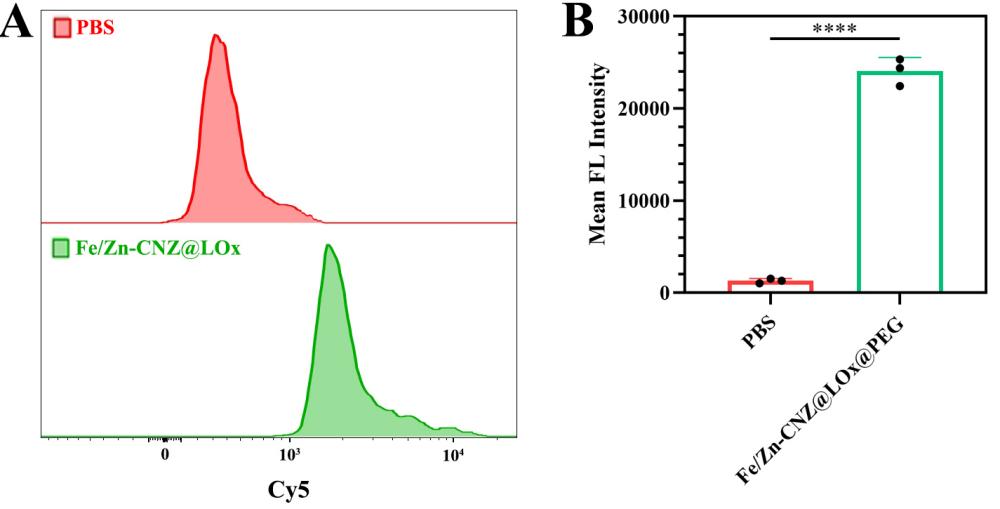


**Figure S5.** The analysis of Fe/Zn-CNZ@LOx@PEG uptake by PCa cells.


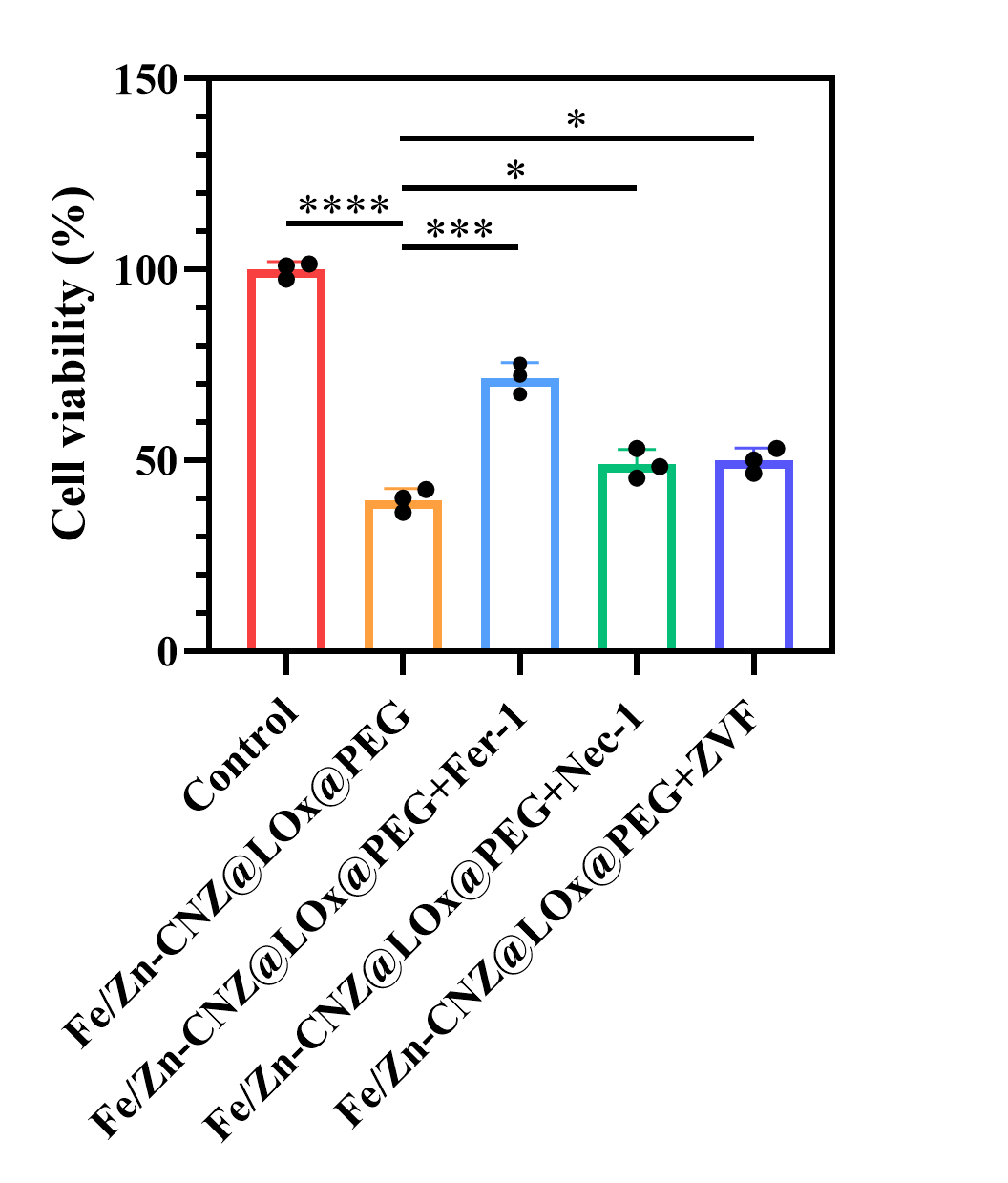


**Figure S6.** The effects of Fer-1, ZVF and Nec-1 on the reduction of proliferation activity of PCa cells induced by Fe/Zn-CNZ@LOx@PEG were investigated.


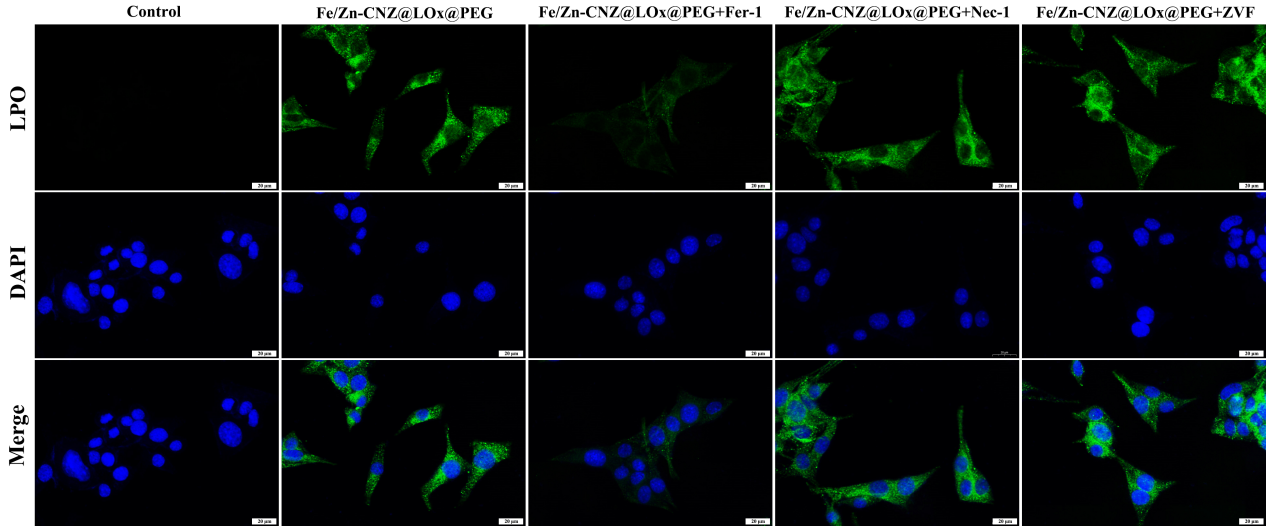


**Figure S7.** The effects of Fer-1, ZVF and Nec-1 on the accumulation of lipid peroxides (LPO) in PCa cells induced by Fe/Zn-CNZ@LOx@PEG.


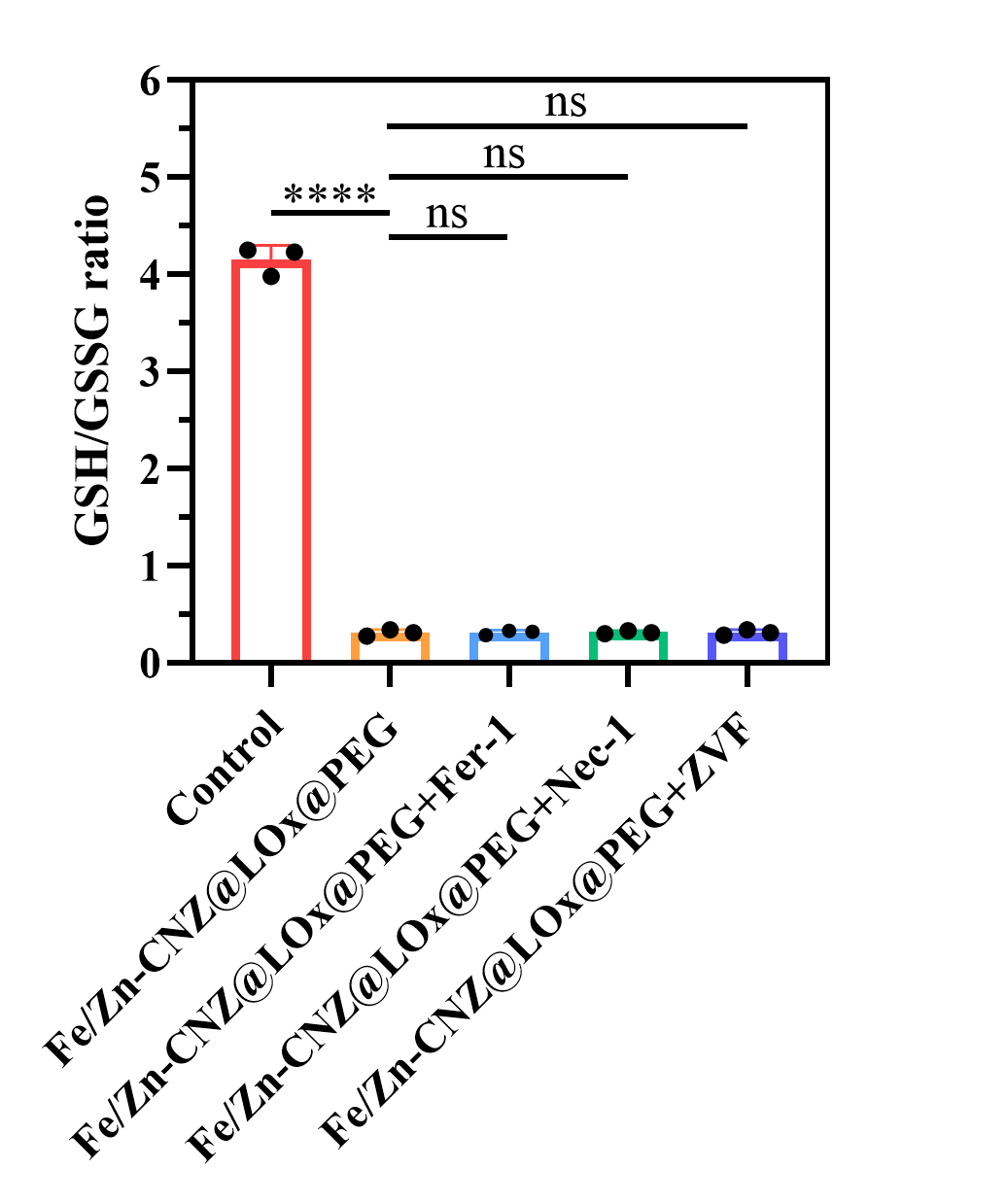


**Figure S8.** The effects of ferroptosis inhibitor Fer-1, apoptosis inhibitor Z-VAD-FMK (ZVF), and necroptosis inhibitor Necrostatin-1 (Nec-1) on the depletion of GSH in PCa cells induced by Fe/Zn-CNZ@LOx@PEG were investigated.
